# Experimental research on the performances of water jet devices and proposing the parameters of borehole hydraulic mining for oil shale

**DOI:** 10.1371/journal.pone.0199027

**Published:** 2018-06-20

**Authors:** Jiwei Wen, Chen Chen, Urso Campos

**Affiliations:** 1 College of Environment and Civil Engineering, Center for Postdoctoral Studies of Geological Resources and Geological Engineering, State Key Laboratory of Geohazard Prevention and Geoenvironment Protection, Chengdu University of Technology, Chengdu, Sichuan, China; 2 Trenchless Technology Center, Louisiana Tech University, Ruston, LA, United States of America; 3 Key Laboratory of Drilling and Exploitation Technology in Complex Conditions, Ministry of Land and Resources, Jilin University, Changchun, Jilin, China; 4 Key Laboratory of Metallogenic Prediction of Nonferrous Metals and Geological Environment Monitoring of Ministry of Education, Central South University, Changsha, Hunan, China; 5 National-Local Joint Engineering Laboratory of In-situ Conversion, Drilling and Exploitation Technology for Oil Shale, Changchun, Jilin, China; 6 College of Construction Engineering, Jilin University, Changchun, Jilin, China; China University of Mining and Technology, CHINA

## Abstract

Oil shale is an unconventional energy source, and it is also a potential petroleum substitute. Nowadays, the energy shortage is becoming more and more prominent, oil shale has attracted the attention of energy researchers all over the world. Borehole hydraulic mining is an effective method to exploit the underground oil shale which has more prominent advantages than other conventional mining methods. Jet devices are the key component of borehole hydraulic mining, which include the straight cone nozzle, organ pipe nozzle and self-excited oscillation pulsed jet nozzle. Also, the reasonable mining parameters are also crucial in mining underground oil shale efficiency. The jet characteristics of the non-submerged water jet, submerged water jet, direct water jet, cavitating water jet, and pulsed water jet are also explained and compared based on theoretical analysis. The jet performance of the non-submerged water jet is better than the submerged water jet. Each type of jet devices has its own basic principles and optimal structural parameters. The best operating scheme of borehole hydraulic mining for underground oil shale is to use the pulsed water jet which is produced by the self-excited oscillation pulsed jet nozzle to break underground oil shale under the non-submerged condition. Moreover, the pulsed water jet should be placed parallel to the oil shale bedding. In addition, under the preconditions of ensuring the safety and reliability of the hydraulic mining equipment and pipelines connection, the jet pressure and jet flow should be raised as much as possible, so as to obtain the much higher mining efficiency. These results and conclusions can provide very valuable guidance for borehole hydraulic mining of underground oil shale.

## Introduction

There is no doubt that energy has always been one of the substances closely bound up with human daily life. The four essential requirements of the human: clothing, food, housing, and transportation are all dependent on energy. In addition, the development of societies, economies, national security, and stability are also inseparable from energy [1]. The unconventional oil and gas resources such as: oil shale, shale gas, methane hydrates, coalbed methane, tight gas sandstone, etc., have been gaining more and more attention worldwide in recent years with the fluctuating global oil prices [2]. Oil shale, in particular, has been widely utilized as a supplement to conventional energy resources because of the large reserves of oil shale, and its applicability in different fields such as: building and chemical materials industries, agriculture, medicine, environmental protection, etc. [3–8].

Currently, there are four main mining methods of oil shale: open cast mining, underground mining, in-situ mining, and borehole hydraulic mining. Among these four mining methods, borehole hydraulic mining technology is the especially suitable for mining deep and low-grade underground oil shale deposits [9–15]. It follows the same basic principles as the high-pressure jet grouting and hydraulic coal mining, they all use high-pressure jet to break the ore bed or the rock and soil mass [14–17]. Moreover, borehole hydraulic mining technology has many advantages. The most significant advantage is that the method does not need to remove the overlying strata. It will bring the obvious benefit is a simple, reliable, and safe operation. Additionally, it is very good for the environmental protection. The basic principle of borehole hydraulic mining was proposed by the American scholar Aye-Kraay Thor in 1932, and also independently proposed by the former Soviet Union scholar Tupicen in 1936. In the beginning of 20th century, the United States carried out the research on borehole hydraulic mining technology, and successfully carried out the mining of uranium deposits, asphaltene sand, granular phosphorite and sandstone, which was subsequently applied in production practice in Russia, Yugoslavia, and other countries [11–16]. In the world, many countries have successfully used borehole hydraulic mining technology to mine coal, iron ore, quartz sand, kimberlite, and other mineral resources. Dimitrijevic proposed some valuable empirical formulas for estimating technological parameters for borehole hydraulic mining [13]. Since September 2009, the Ministry of Education and the Ministry of Finance of China have commissioned Jilin University to take the lead, and combined 16 dominant units in the field of oil shale resources research in China, in order to effectively solve the major problems in the national economic and social development, to promote the innovation an the deep cooperation between industry, education and research, and to enhance the ability of universities to serve the major national demand. The project of the national leap plan “National Potential Oil and Gas Resources (Exploration and Utilization of Oil Shale), Cooperation and Innovation for Production, Education and Research” was launched and implemented [14]. Yan Xuanchen tested the physical and mechanical parameters of the Nong’an oil shale and gave some valuable suggestions for borehole hydraulic mining [15]. Zhao Guijie used the FLAC 3D software to simulate the best mining spacing in the Nong’an oil shale mining area and proposed the optimal mining hole spacing and the distribution regularities of the vertical displacement of the surrounding rock near the excavation and earth surface [16]. Bondarchuk presented the technological processes of borehole hydraulic mining for pay zone development [17].

Actually, the process of borehole hydraulic mining for underground oil shale usually includes three key steps: (1) the borehole is drilled through the underground oil shale bed from the ground; (2) a hydraulic monitor and other hydraulic or pneumatic mining equipment is placed down into the borehole from the ground; the high-pressure mud pump is turned on and water is pumped from the water tank on the ground. The high-pressure water flows downward along the central hole of the hydraulic monitor. The jet device is connected to the end of the hydraulic monitor so the high-pressure water jet is ejected through the jet device so that it can break the underground oil shale into several small pieces and peel them off from the parent rock to produce a mixed solid-liquid; flowable ore pulp of oil shale at the bottom of the borehole; (3) the ore pulp of oil shale is lifted from the bottom to the surface using a pneumatic or hydraulic lifting device.

As mentioned above, the most critical step in the entire borehole hydraulic mining process is breaking the underground oil shale by the high-pressure water jet produced by the jet device. The outcome of this step will directly determine the success and efficiency of the mining process [14–18]. Additionally, in this study, the straight-cone nozzle, self-resonating cavitating jet nozzle, and self-excited oscillation pulsed jet nozzle are collectively referred to as the jet devices.

In conclusion, the jet devices are the most critical component in the process of borehole hydraulic mining. Consequently, their jet performance will directly influence the mining outcome for the underground oil shale. Moreover, the mining parameters also play crucial roles in the mining process [12–18]. Therefore, this study aims to compare the jet performances of the different jet devices, and propose the parameters for borehole hydraulic mining of Huadian oil shale in China.

## Theories of high pressure water jet

High-pressure water jet technology uses water as the work medium. The high-pressure energy of water is attained by using high-pressure pumps. Furthermore, the energy concentrated liquid column, that is the high-pressure water jet, is ejected from the outlet of a nozzle at high speed after the high-pressure water continuously flow past the internal flow channel of the nozzle [15–27]. Some of the many advantages of using a nozzle include: cleanliness, concentrated cutting energy, consistency in water jet output, and reduction of sparks and sharp rubbles resulting in a safe cutting process. In addition, it can also be combined with a numerical control machine tool to precisely cut arbitrary and complex shapes. High-pressure water jet technology has been widely used in many industry fields such as: coal, metallurgy, petroleum, municipal, etc. Some of its specialized applications include: mining, drilling, cleaning, surface treating, industrial cutting, etc. Conclusively, it has resulted in many remarkable social and economic benefits.

### Comparison of non-submerged water jet and submerged water jet

The water jet can be divided into non-submerged water jet and submerged water jet according to the difference between the jet medium and the surrounding [21–25]. When the density of water is much higher than the density of the surrounding, the water jet is categorized as a non-submerged water jet (i.e. water jet spraying into the air). However, when the density of water is similar to the density of the surrounding, the water jet is categorized as a submerged water jet (i.e. water jet spraying into the water).

**Fig 1:**
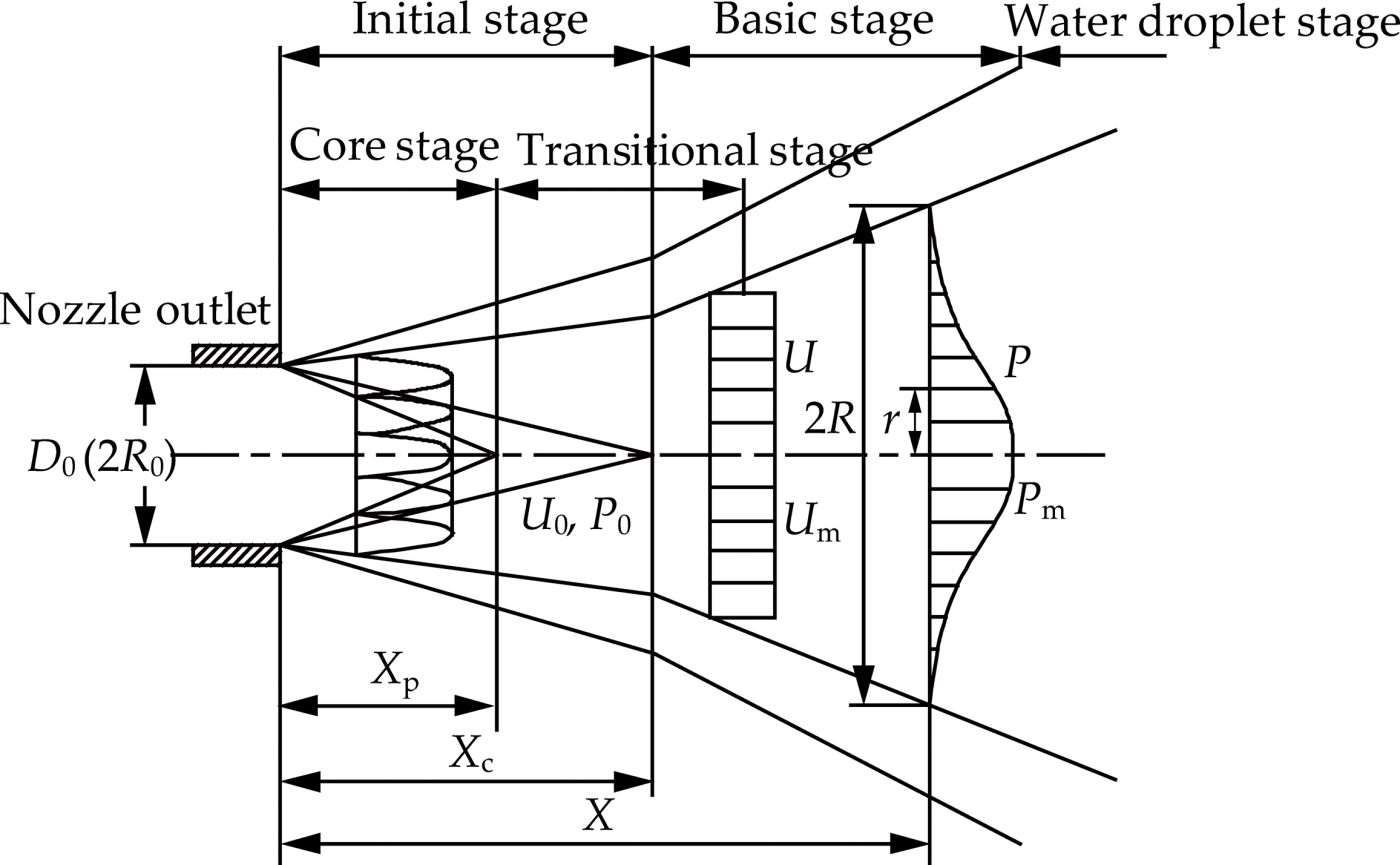
Schematic diagram of the non-submerged water jet.

The characteristics of the non-submerged water jet mainly include the length of the initial stage and the diffusion laws. Fig 1 presents a schematic diagram of the non-submerged water jet. In the spray direction of the water jet, the water jet can be divided mainly into three different stages: the initial stage, the basic stage, and the water droplet stage. The length of the initial stage includes the length of the core stage and the partial length of the transitional stage. The basic stage is also known as the breaking stage. During the core stage, in the initial stage, the water jet closes in to the nozzle outlet which is at its smallest cross-sectional area. The velocity of each point in its cross section is equal to the jet velocity at the nozzle outlet.

Due to the frictional resistance between the surface of the water jet and the air, ripples appear on the surface of the water jet. Their amplitude is proportional to the distance away from the nozzle outlet. When the amplitude reaches a certain degree, the surface of the water jet begins to break. This is due to small amounts of air inhaled resulting in the surface of the water jet breaking the water mass or dispersing it away. The extent of the core stage is inversely proportional to the distance away from the nozzle outlet. When certain distance is reached, the core stage disappears completely. In the basic stage, as the amount of inhaled air in the water jet gradually increases, the phenomenon accelerates resulting in the water mass on the surface of the water jet to burst into droplets. As the water continues to disperse, the center of the water jet breaks from the previous tightened status, and the water mass gradually disperses with the increase of the distance to the nozzle outlet and eventually it transforms into water droplets. The following conclusion was reached after numerous experiments [21–25].

First, when the Reynolds number (Re) is greater than 2×10^5^, the length of the initial stage (*x*_c_) is only closely related to the machining quality and the flow channel’s shape of the nozzle but has no relation with the injection pressure. Usually, the length of the initial stage (*x*_c_) is 65–135 times the diameter of the nozzle outlet (*d*).

Second, in the basic stage, the diffusion of water jet is relatively stable. Its diffusion complies with the following two diffusion law equations [21–25]:

$$R=k\cdot \sqrt{x}$$ (1)

$$\frac{R}{R_{0}}=k_{1}\cdot \sqrt{\frac{x}{R_{0}}}$$ (2)

Where, *R* is the radius of the water jet section, *R*_0_ is the radius of the nozzle outlet, *k* is the coefficient associated with the nozzle, *k*_1_ is 0.12–0.18, *x* is the distance to the nozzle outlet.

Third, in the non-submerged water jet, the dynamic pressure can represent the kinetic energy of the fluid in a unit time. In addition, the dynamic pressure can also reflect the change of the air intake and the velocity attenuation regularity. The dynamic pressure of the fluid at the nozzle outlet complies with the following equation [21–25]:

$$P_{0}=\frac{1}{2}\rho \cdot U_{0}^{2}=\frac{1}{2}\rho \cdot c^{2}\cdot \frac{2P_{i}}{\rho }=c^{2}\cdot P_{i}$$ (3)

Where, *P*_0_ is the dynamic pressure of the fluid at the nozzle outlet, *ρ* is the density of water, *U*_0_ is the initial jet velocity of the water jet, *P*_*i*_ is the dynamic pressure of the fluid at the nozzle inlet, *c* is the velocity coefficient of the nozzle.

Because the velocity coefficient (*c*) is usually a constant less than 1, it is clear that the dynamic pressure of the fluid at the nozzle outlet (*P*_0_) is lower than the dynamic pressure of the fluid at the nozzle inlet (*P*_*i*_) from the above equation. This phenomenon is mainly due to the energy loss caused by the fluid flow past the nozzle channel.

The pressure distribution of the water jet in the basic stage complies with the following equation [21–25]:

$$\frac{P}{P_{m}}=f(\eta )={\left(1-\eta^{1.5}\right)}^{2}$$ (4)

Where, *P* is the dynamic pressure of the any point at the water jet section, *P*_m_ is the dynamic pressure on the axial center of the water jet section, *η* is the dimensionless radial distance.

$$\eta =\frac{y}{R}$$ (5)

Where, *y* is the radial distance from the water jet axis.

The dynamic pressure on the axial center of the water jet stays the same in the region of the core stage, it only begins to drop when it exceeds the core stage. The law of the dynamic pressure on the axial center of the water jet complies with the following equation [21–25]:

$$\frac{P_{m}}{P_{0}}=\left\{ \begin{array}{l} 1,\;X\leq X_{c}\\ \frac{X_{c}}{X},\;X>X_{c} \end{array}\right.$$ (6)

Where, *X* is the distance of from any point on the axial center of the water jet from the nozzle outlet.

The length of the initial stage (*X*_c_) complies with the following equation [21–25]:

$$\left\{ \begin{array}{l} X_{c}=3.89{\left(\frac{R_{0}}{k}\right)}^{2}\\ {\bar{X}}_{c}=\frac{X_{c}}{R_{0}}=3.89\frac{R_{0}}{k^{2}}\\ {\bar{R}}_{c}=\frac{R_{c}}{R_{0}}=1.97 \end{array}\right.$$ (7)

Where, ${\bar{X}}_{c}$ is the dimensionless length of the initial stage of the water jet, ${\bar{R}}_{c}$ the dimensionless radius of the water jet in the initial stage, *R*_c_ is the radius of the water jet at the end of the initial stage.

**Fig 2:**
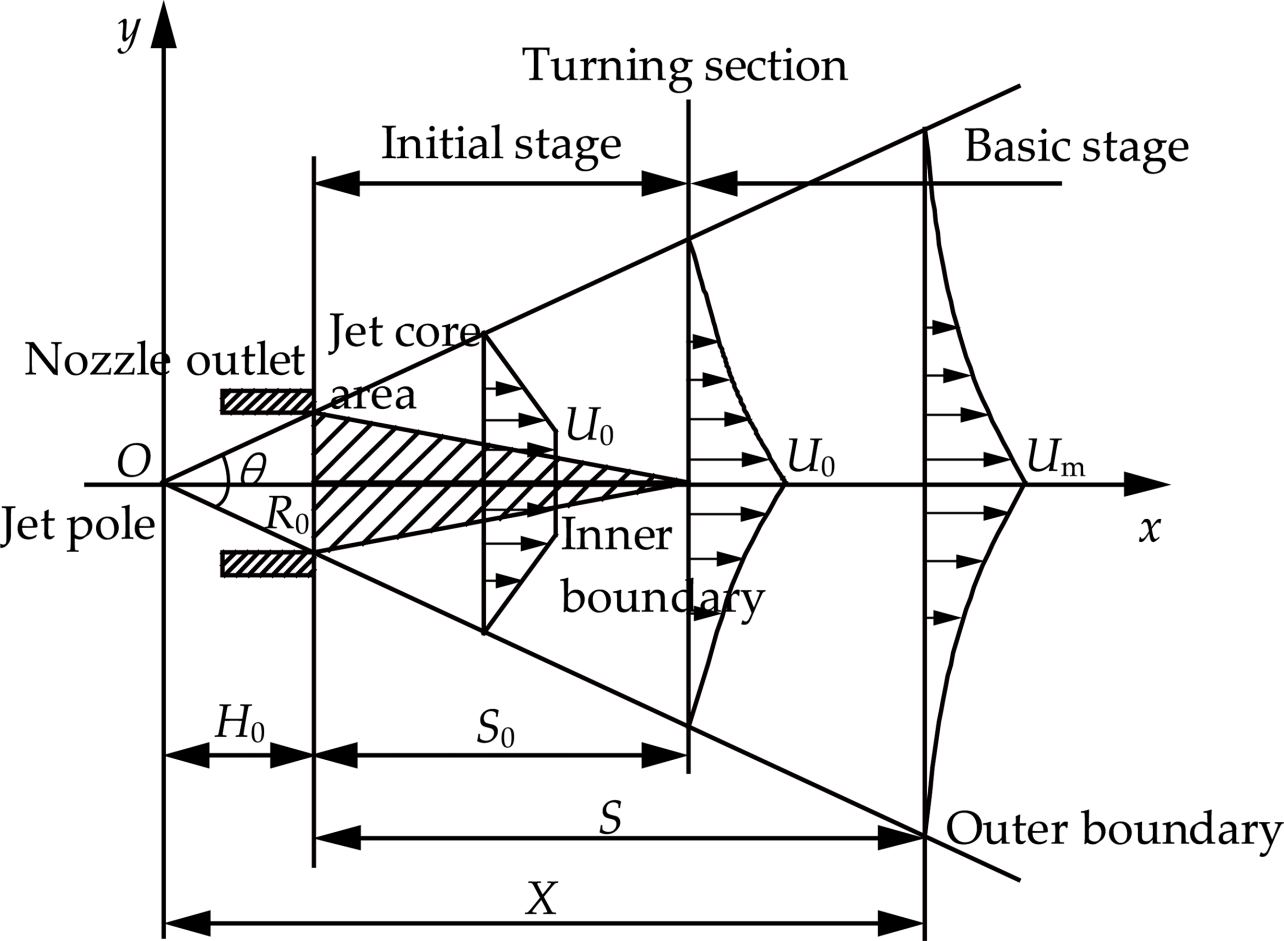
Schematic diagram of the submerged water jet.

Fig 2 presents a schematic diagram of the submerged water jet. For the submerged water jet, the jet velocity is uniform at the nozzle outlet. However, when the water jet leaves the nozzle outlet, it sucks the water in the surrounding environment, which will gradually reduce the jet velocity of the water jet and widen the jet boundary. Meanwhile, the core area of the water jet shrinks. Usually, the boundary of the water jet velocity is zero at the outer boundary. The water jet maintains the initial jet velocity (*U*_0_) in the inner boundary. However, the water jet velocity on the jet axis gradually reduces after the turning section, that is the initial stage of the water jet.The area between the inner boundary and the outer boundary is the boundary layer. The jet section which locates the crossing point of the inner boundary and the jet axis (*x*) is the turning section. The crossing point of the reverse extension of the outer boundary is the jet pole. *X* is the distance of from any point on the axial center of the water jet from the jet pole.

The law of the velocity distribution inside the boundary layer of the submerged water jet complies with the following equation [21–25]:

$$\frac{U}{U_{m}}={\left(1-\eta^{1.5}\right)}^{2}$$ (8)

Where, *U* is the velocity of the water jet, *U*_m_ is the velocity on the jet axis of the water jet, *η* is the dimensionless radial distance.

The jet radius at the turning section of the submerged water jet (R*) complies with the following equation [21–25]:

$$R^{*}=3.3R_{0}$$ (9)

Where, *R*_0_ is the radius of the nozzle outlet.

The length of the core stage of the submerged water jet (*S*_0_) complies with the following equation [21–25]:

$$S_{0}=2.3H_{0}=9.12R_{0}$$ (10)

Where, *H*_0_ is the distance between the jet pole and the nozzle outlet.

The attenuation law of the axial velocity of the submerged water jet complies with the following equation [21–25]:

$$\frac{U_{m}}{U_{0}}=\left\{ \begin{array}{l} 1,\;S\leq S_{0}\\ \frac{13.2R_{0}}{S+4R_{0}},\;S>S_{0} \end{array}\right.$$ (11)

Where, *S* is the distance from any point in the water jet to the nozzle outlet.

In addition, Hu Yi proposed a theory of impinging jet: the submerged impinging jet mainly includes three regions: the free jet region, stagnation region, and wall jet region. Moreover, according to the experimental research, the SC-CO_2_ jet generally has a similar structure as a water jet, and the relatively lower viscosity of the SC-CO_2_ jet can help to reduce the amount of energy dissipated [26].

Through above mentioned about the non-submerged water jet and the submerged water jet, the comparison of the main jet performances of these two water jets is shown in Table 1. The representation of each symbol in Table 1 are consistent with the above mentioned.

**Table 1:** Comparison of the main jet performances of non-submerged and submerged water jet.

Jet Performances	Jet Types	
	Non-submerged water jet	Submerged water jet
The axial dynamic pressure attenuation in the basic stage	$\frac{P_{m}}{P_{0}}=X_{c}$, $\frac{1}{X}=\frac{X_{c}}{X}$	$\frac{P_{m}}{P_{0}}\propto \frac{1}{X^{2}}$
The axial velocity attenuation in the basic stage	N/A	$\frac{U_{m}}{U_{0}}=\frac{13.2R_{0}}{X}$
The velocity distribution of the jet section in the basic stage	N/A	$\frac{U}{U_{m}}={\left(1-\eta^{1.9}\right)}^{2}$
The dynamic pressure distribution of the jet section in the basic stage	$\frac{P}{P_{m}}={\left(1-\eta^{1.5}\right)}^{2}$	$\frac{P}{P_{m}}={\left(1-\eta^{1.5}\right)}^{4}$
The diffusion of the water jet	$R=k\sqrt{x}$, *R*_*c*_ = 1.97*R*_0_	R* = 3.3*R*_0_
The length of the core stage	*X*_c_ = (65–135) *D*_0_	*S*_0_ = 9.12 *R*_0_
The effective jet distance	100–150 *D*_0_	5–8 *D*_0_

Few remarks are concluded based on above mentioned as follows: First, the length of the core stage and the effective jet distance of the non-submerged water jet is much longer than the submerged water jet. Second, the distribution of the velocity and the dynamic pressure at the water jet section of the non-submerged water jet is relatively uniform and the change is small. Third, the diffusion rate of the water jet of the submerged water jet is faster than the non-submerged water jet, so the energy dissipation in the submerged water jet is fast and the effective jet distance is short. Finally, the attenuation of the axial dynamic pressure and the axial velocity of the submerged water jet is faster than the non-submerged water jet, so the efficiency of the submerged water jet on breaking or cutting materials is greatly influenced by the jet distance.

Therefore, the non-submerged water jet results in higher efficiency when breaking oil shale than the submerged water jet. Conclusively, it is more effective to select the non-submerged water jet in the borehole hydraulic mining for oil shale.

### Comparison of the direct water jet, cavitating water jet and pulsed water jet

#### The characteristics of the direct water jet

The direct water jet is the simplest and most common form of water jet. It is always a continuous water jet. Park fountains, sprinkler for watering plants, and fire extinguishers are all typical applications of the direct water jet. The above water jet descriptions of the non-submerged water jet and the submerged water jet are particularly suitable to describe the jet performance of the direct water jet. There are many types of nozzle that can produce the direct water jet such as: the straight cone nozzle, conical nozzle, arc inlet nozzle, elliptical inlet nozzle, etc. However, based on comprehensive considerations such as: machining processes, working efficiency, applicability condition, and costs; it is concluded that the best and the most widely used nozzle is the straight cone nozzle [14,18–25].

#### The characteristics of the cavitating water jet

The cavitating water jet uses cavitation bubbles to reinforce the water jet effect on breaking or cleaning materials. Usually, the cavitation bubbles are formed in submerged condition using natural or artificial means to produce a low-pressure area which is typically less than the local saturated vapor pressure of the water at the corresponding temperature [21,23,28–29]. The relationship of the pressure impact between the cavitating water jet and the direct water jet complies with the following equation [21,23]:

$$P_{i}=\frac{P_{s}}{6.35}\exp\left(\frac{2}{3}\alpha \right)$$ (12)

Where, *P*_*i*_ is the impact pressure of the cavitating water jet, *P*_*s*_ is the impact pressure of the direct water jet, *α* is the volume fraction of the gas in the water.

The volume fraction of the gas in the water (*α*) can be obtained by a series of experiments. When volume fraction of the gas in the water (*α*) is 0.1–0.17, the relationship between the impact pressure of the cavitating water jet (*P*_*i*_) and the impact pressure of the direct water jet (*P*_*s*_) complies with the following equation [21,23]:

$$P_{i}=(8.6∼124)P_{s}$$ (13)

Consequently, the impact pressure of the cavitating water jet (*P*_*i*_) is 8.6–124 times the impact pressure of the direct water jet (*P*_*s*_).

There are four main cavitating erosion mechanisms described by the following theories: the theory of mechanical action, the theory of chemical corrosion, the theory of electrochemical action, and the theory of thermal action [21,23]. Among these theories, the theory of mechanical action is generally accepted by the public. The theory of mechanical action presents the microjets and shock waves which are generated by the burst of cavitation bubbles as the cause of cavitating erosion on the materials’ surface. The gas content in the water jet beam and the jet distance are the main influencing factors of the micro-jets which are formed by cavitation bubbles and the cavitating erosion on the materials’ surface. If the jet distance is too short, the cavitation bubbles in the cavitating water jet beam may not fully develop and lead to weak cavitating erosion effects. However, if the jet distance is too long, although the cavitation bubbles fully develop, they may burst before they reach the materials’ surface. Therefore, the location of the nozzle and the materials’ surface should be at an optimum distance from each other so that the zone of cavitation bubbles burst at the desired location. In addition, there is another theory of cavitating erosion that the pressure fluctuation is the main cause of the cavitating erosion [28]. In the process of the cavitating water jet, sometimes the water hits the materials’ surface, and sometimes the cavitation bubbles hit the materials’ surface. Due to the difference in density between the water and the cavitation bubbles, the action of pressure fluctuation is generated on the materials’ surface.

Generating cavitation in the water jet beam is the key to cavitating water jet [21,23,28–29]. There are six main types of nozzles used for in cavitating water jet: organ pipe nozzle, Helmholtz nozzle, central body nozzle, angular nozzle, rotary vane nozzle, and radial flow venturi nozzle. In addition, the cavitating water jet can also be generated by means of artificial water flooding and ultrasonic waves. However, the organ pipe nozzle is the most widely used to produce the cavitating water jet [21,23,29].

#### The characteristics of the pulsed water jet

The pulsed water jet is different from the conventional continuous direct water jet. Its jet energy and flow parameters change with time and its striking force also changes periodically, resulting in an unstable water jet. The effect on breaking or cleaning materials of the pulsed water jet is mainly determined by the effect of water hammer pressure. There are three main ways to get the pulsed water jet: excitation, blocking, and squeezing [21]. Among them, the blocking and squeezing generally have some disadvantages including that the effective utilization rate of energy is low, energy dissipation is large, the equipment wear is more severe, and an extra high-pressure dynamic seal needs to be installed. On the other hand, the excited pulsed water jet uses the principles of rheology and transient to change the flow structure of the continuous direct water jet to produce the pulsed water jet based on a periodic change. There are three different types of the excited pulsed water jet: self-excited pulsed water jet, modulating pulsed water jet, and resonant pulsed water jet. It is a common engineering practice to combine these three different types of excited pulsed water jet to improve the striking effect of the pulsed water jet. The most typical example is the self-excited oscillation pulsed jet nozzle.

### Basic principles and design principles of jet devices

The basic principles and the design principles of the jet devices are very important to design the structure of high efficiency nozzles [14,18,21–25]. There are three kinds of nozzle with different structures: straight cone nozzle which can produce a continuous direct water jet; the organ pipe nozzle which can produce a self-resonating cavitating water jet; and the self-excited oscillation pulsed jet nozzle which can produce a pulsed water jet. Considering the dimensions of high-pressure pipe and the machining method of these three nozzles, in this study the parameters of these nozzles’ inlet diameter (*D*) are all set to 19 mm. Moreover, the access section of these nozzles is connected to the high-pressure pipe. The length of the access section (*l*’) has no direct effect on the jet performances of these nozzles, and its value is based on the suitable for matching the length of the connecting section of the high-pressure pipeline. The length of the access section (*l*’) of these three different nozzles is 32 mm. In addition, all the nozzles in this study are made of the stainless steel 304 by comprehensive considering the aspects of nozzle manufacturing, cost, service life, etc.

#### Basic principle and design principle of the straight cone nozzle

**Fig 3:**
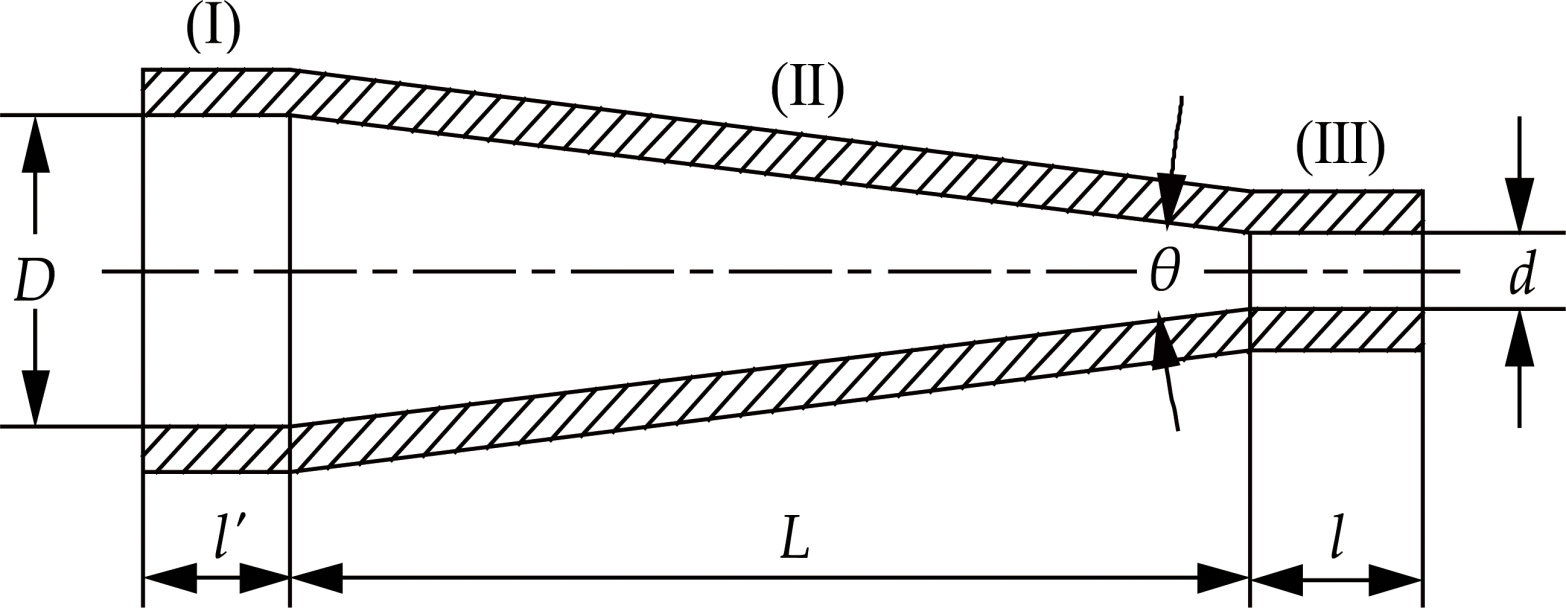
Schematic diagram of the straight cone nozzle structure.

The straight cone nozzle is a simple structure and highly efficient nozzle with an excellent jet performance. Apart from its flow coefficient that can reach as high as 0.98, it can be used under an injection pressure ranging from 10–60 MPa (maximum pump pressure), which is exactly the driving pressure range of the water jet during the hydraulic mining process. Therefore, it is often used in hydraulic mining operations [14,18,21–25]. Fig 3 shows a schematic of the straight cone nozzle structure. It can be divided into three parts: the access section (I), the contraction section (II), and the exit section (III).

The structural design of the straight cone nozzle complies with the following equation [14,18,21–22]:

$$\frac{D-d}{L-l}=2\tan\frac{\theta }{2}$$ (14)

Where, *D* is the inlet diameter, *d* is the outlet diameter, *L* is the length of the contraction section, *l* is the length of the exit section, and *θ* is the contraction angle.

Considering the cost of nozzle manufacturing, the results of the orthogonal experimental design, range analysis, and variance analysis comprehensively, the optimal structural parameters of the straight cone nozzle can be obtained and listed them in Table 2. The details can be seen in reference [14,18]. Therefore, the total length of the straight cone nozzle is composed of the length of the access section (*l*’), the length of the exit section (*l*), and the length of the contraction section (*L*) for a total of 54.99 mm.

**Table 2:** Optimal structural parameters of the straight cone nozzle.

Serial Number	Structural Parameters	Values
1	Inlet diameter (*D*)	19 (mm)
2	Length of the access section (*l*’)	32 (mm)
3	Outlet diameter (*d*)	4 (mm)
4	Contraction angle (*θ*)	60 (°)
5	Length to diameter ratio (*l*/*d*)	2.5
6	Length of the exit section (*l*)	10 (mm)
7	Length of the contraction section (*L*)	12.99 (mm)

#### Basic principle and design principle of the organ pipe nozzle

**Fig 4:**
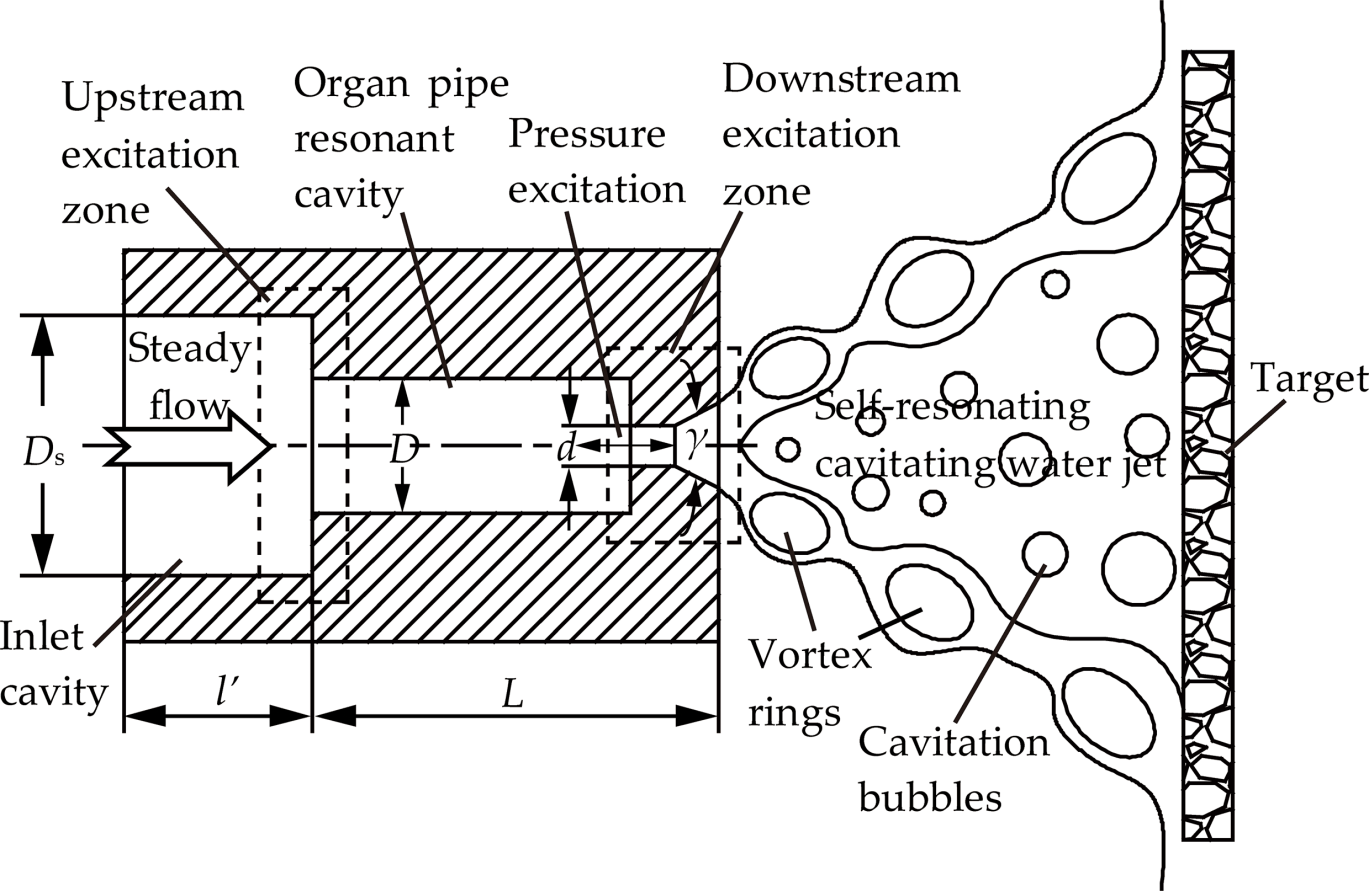
Schematic diagram of the self-resonating cavitating jet nozzle structure and operating principle.

The organ pipe nozzle is a typical self-resonating cavitating jet nozzle based on the theory of resonance. The resonance of the high-pressure water in the cavity of the nozzle is generated by its self-excited excitation. Fig 4 presents a schematic diagram of the self-resonating cavitating jet nozzle structure and operating principle. It uses an organ-pipe like resonant cavity as excitation amplifier. The length of the resonant cavity is *L*, and the diameter of the resonant cavity is *D*. The upper end of the resonant cavity is connected to the inlet cavity. The diameter of the inlet cavity is *D*_s_. The contraction section of the resonant cavity inlet is (*D*_s_/*D*)^2^. The lower end of the resonant cavity is connected to the nozzle outlet section. The diameter of the nozzle outlet is *d*. In addition, the diffusion angle of the nozzle outlet (*γ*) usually needs to be designed. It is easy to form the boundary layer separation because the diffusion angle of the nozzle outlet (*γ*) can decrease the axial jet velocity and increase the pressure gradient at the nozzle outlet. This outcome we desired as it generates vortexes and shedding. In addition, it helps to induce a strong resonance to promote the effect of the cavitation erosion. The contraction section of the resonant cavity outlet is (*D*/*d*)^2^. These two contraction sections of the resonant cavity are the mechanisms of the self-resonating and feedback. When the steady flow enters the internal cavity of the organ pipe nozzle from the inlet, the contraction sections not only can induce the initial pressure surge of the liquid flow, but also can return the pressure surge to the resonant cavity, which can cause feedback pressure excitation. Per theories of transient flow, if the natural frequency of the organ pipe resonant cavity matches the pressure excitation frequency, the feedback pressure excitation will be amplified resulting in the appearance of the flow resonance in the organ pipe resonant cavity to form a standing wave. At this stage, the eddies in the jet shear layer will be transformed into large scale separated ring vortexes, which can enhance the cavitation erosion effect of the water jet. It is always used underwater to further enhance its cavitation effect [21,23].

The designed range of the optimal structural parameters of the organ pipe nozzle are listed in Table 3. The Strouhal Number (Sr*) should be as small as possible in the process of designing the organ pipe nozzle [21,23].

**Table 3:** The designed range of the optimal structural parameters of the organ pipe nozzle.

Serial Number	Structural Parameters	Values
1	The contraction section of the resonant cavity inlet (*D*_s_/*D*)^2^	3.5–4.5
2	The contraction section of the resonant cavity outlet (*D*/*d*)^2^	10–11
3	Mach number (Ma)	0.08–0.09
4	Strouhal Number (Sr)	0.3

When the natural frequency (*f*) equals the critical self-resonating frequency (*f* *), the strongest resonance takes place in the organ pipe nozzle. The natural frequency of the organ pipe resonant cavity (*f*) complies with the following equation [21, 23]:

$$f=K_{N}\cdot \frac{a}{L}$$ (15)

Where, *K*_N_ is the modulus of coefficient (*K*_N_ complies with the Eq 16), *a* is the disturbance wave velocity of the organ pipe resonant cavity, *L* is the length of the organ pipe resonant cavity.

$$K_{N}=\left\{ \begin{array}{l} \frac{2N-1}{4},\;\left[{\left(\frac{D_{s}}{D}\right)}^{2}>>1,\;and\;{\left(\frac{D}{d}\right)}^{2}>>1\right]\\ \frac{N}{2},\;\left[{\left(\frac{D_{s}}{D}\right)}^{2}>>1,\;but\;{\left(\frac{D}{d}\right)}^{2}<<c\right] \end{array}\right.$$ (16)

Where, *N* is the oscillation modulus in the organ pipe resonant cavity (N = 1,2,3…), *c* is a constant determined by the experiment.

In general, the critical self-resonating frequency of the organ pipe nozzle (*f* *) complies with the following equation [21, 23]:

$$f^{*}=Sr^{*}\cdot \frac{\mathrm{Ma}}{d}\cdot a$$ (17)

Where, Sr* is the critical Strouhal Number (Sr* complies with the Eq 18), Ma is the Mach number (Ma complies with the Eq 19).

$$Sr^{*}=f^{*}\cdot \frac{d}{u}$$ (18)

Where, *u* is the jet velocity of the organ pipe nozzle outlet.

$$\mathrm{Ma}=\frac{u}{a}$$ (19)

In order to get the strongest resonance, the structural parameters of the organ pipe resonant cavity should comply with the following equation [21, 23]:

$$\frac{L}{d}\approx \frac{K_{N}}{\mathrm{Ma}\cdot Sr^{*}}$$ (20)

As discussed above, the optimal structural parameters of the organ pipe nozzle are listed in Table 4. Therefore, the total length of the organ pipe nozzle equals the length of the access section (*l*’) plus the length of the resonant cavity (*L*), it is 61.4 mm.

**Table 4:** Optimal structural parameters of the organ pipe nozzle.

Serial Number	Structural Parameters	Values
1	Inlet diameter (*D*_s_)	19 (mm)
2	Length of the access section (*l*’)	32 (mm)
3	Resonant cavity diameter (*D*)	10 (mm)
4	Resonant cavity length (*L*)	29.4 (mm)
5	Outlet diameter (*d*)	3.1 (mm)
6	Outlet diffusion angle (*γ*)	60 (°)

#### Basic principle and design principle of the self-excited oscillation pulsed jet nozzle

**Fig 5:**
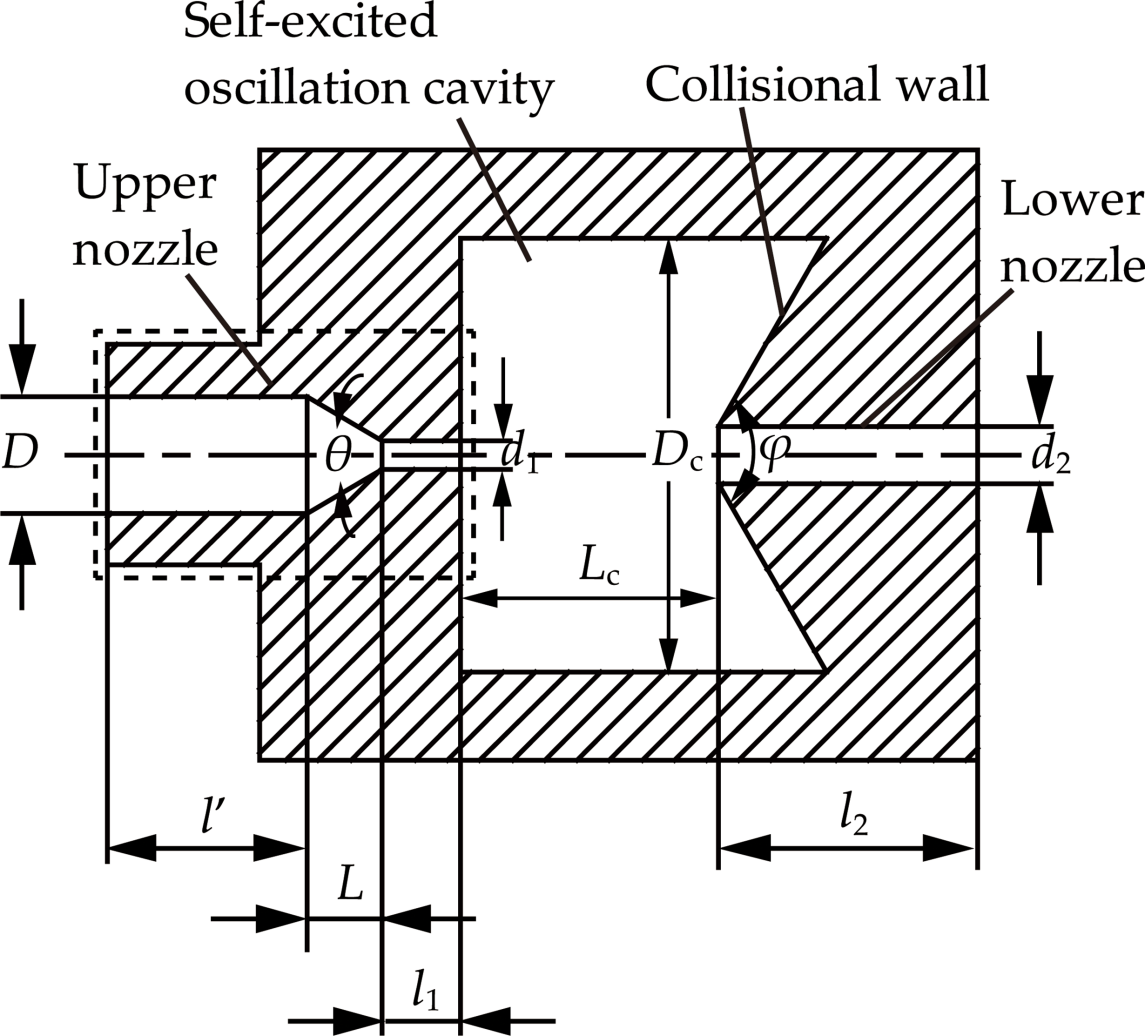
Schematic diagram of the self-excited oscillation pulsed jet nozzle structure.

The self-excited oscillation pulsed jet nozzle is a typical pulsed water jet nozzle based on the Helmholtz Oscillation Principle. Some of its advantages are: the structure is relatively simple, no additional driving equipment or structure is attached, no dynamic seal is needed, and almost no moving parts are required. Therefore, it has a high security and reliability in the operation process. Under the same experimental conditions, many experiments show that the self-excited oscillation pulsed water jet nozzle can produce the stagnation pressure of more than 0.25 times the continuous direct water jet, and an instantaneous striking force peak of 1.3–2 times the continuous direct water jet [21–22,30]. Fig 5 presents a schematic diagram of the self-excited oscillation pulsed jet nozzle structure. The self-excited oscillation pulsed jet nozzle mainly consists of the upper nozzle, self-excited oscillation cavity, collisional wall, and lower nozzle. In this study, the upper nozzle is designed as a straight cone nozzle. The process of the pulsed water jet by the self-excited oscillation pulsed jet nozzle begins with the steady and continuous flow into the self-excited oscillation cavity, and then it impacts on the downstream collisional wall. This action creates a pressure disturbance for the steady and continuous fluid to produce the pulsed water jet.

**Fig 6:**
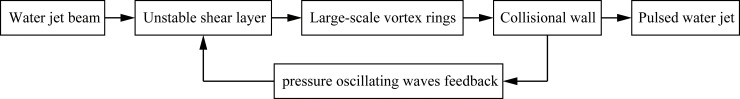
The process of the self-excited oscillation pulsed water jet formation.

Lai SQ and Liao ZF proposed the mechanism of the pulsed water jet production by self-excited oscillation pulsed jet nozzle [30]. The water jet beam is ejected by the upper nozzle outlet at high velocity towards downstream. The vorticity disturbance within a certain frequency in the water jet beam can be amplified. However, the large-scale vortex rings are generated at the shear layer. When these vortex rings collide with the collisional wall, the pressure oscillating waves will be generated in the collision zone, and then reflected to the upstream region at sonic speed. Moreover, it causes a new disturbance again. If the frequency of the new disturbance at this time matches with the frequency of the oscillating wave in the collision zone, and their phase relationship is inverting, this will cause the resonance to be generated. Therefore, there is a reciprocating cycle with the following steps: the vorticity disturbance → amplification → generating new vorticity pulse. In this way, a strong self-excited oscillation pulsed water jet can be generated. Fig 6 shows the process of the self-excited oscillation pulsed water jet formation.

The main structural parameters of the self-excited oscillation pulsed jet nozzle include that the upper nozzle outlet diameter (*d*_1_), the diameter of the self-excited oscillation cavity (*D*_c_), the distance from the upper nozzle outlet to the lower nozzle inlet (*L*_c_), the cone angle of the collisional wall (*φ*), and the lower nozzle diameter (*d*_2_) [21]. The designed range of the optimal structural parameters of the self-excited oscillation pulsed jet nozzle are listed in Table 5.

**Table 5:** The designed range of the optimal structural parameters of the self-excited oscillation pulsed jet nozzle.

Serial Number	Structural Parameters	Values
1	The ratio of the self-excited oscillation cavity diameter to lower nozzle diameter (*D*_c_/*d*_2_)	6–8
2	The ratio of the distance from the upper nozzle outlet to the lower nozzle inlet to the diameter of the self-excited oscillation cavity (*L*_c_/*D*_c_)	0.4–0.7
3	The ratio of the lower nozzle diameter to the upper nozzle outlet diameter (*d*_2_/*d*_1_)	1.6–2.3

As discussed above, the optimal structural parameters of the self-excited oscillation pulsed jet nozzle are listed in Table 6. Therefore, the total length of the self-excited oscillation pulsed jet nozzle is made up of the upper nozzle’s access section (*l*’), the length of the upper nozzle’s contraction section (*L*), the length of the upper nozzle’s exit section (*l*_1_), the distance of from the upper nozzle outlet to the lower nozzle inlet (*L*_c_), and the length of the lower nozzle (*l*_2_) for a total of 143.99 mm.

**Table 6:** Optimal structural parameters of the self-excited oscillation pulsed jet nozzle.

Serial Number	Structural Parameters		Values
1	Upper nozzle	Inlet diameter (*D*)	19 (mm)
2		Length of the access section (*l*’)	32 (mm)
3		Length of the contraction section (*L*)	12.12 (mm)
4		Outlet diameter (*d*_1_)	5 (mm)
5		Length to diameter ratio (*l*_1_/*d*_1_)	2.5
6		Length of the exit section (*l*_1_)	12.5 (mm)
7		Contraction angle (*θ*)	60 (°)
8	Self-excited oscillation cavity	The diameter of the self-excited oscillation cavity (*D*_c_)	70 (mm)
9	Lower nozzle	lower nozzle diameter (*d*_2_)	10 (mm)
10		The cone angle of the collisional wall (*φ*)	120 (°)
11		Length of the lower nozzle (*l*_2_)	45.37 (mm)
12		The distance from the upper nozzle outlet to the lower nozzle inlet (*L*_c_)	42 (mm)

## Experimental study of the water jet performances and the breaking oil shale efficiencies of the jet devices

The water jet performance can be evaluated by its striking force. Under the same experimental conditions, the greater the striking force, the better the water jet performance of the nozzle. Moreover, the breaking of oil shale not only can be used to evaluate the water jet performance of the nozzle, but also can be used to propose the parameters of the borehole hydraulic mining for oil shale. Therefore, the aim of this experimental study focuses on the comparison of the striking forces and the breaking oil shale effects of three different nozzles. (dx.doi.org/10.17504/protocols.io.ppfdmjn)

### The self-developed multifunctional experimental device

**Fig 7:**
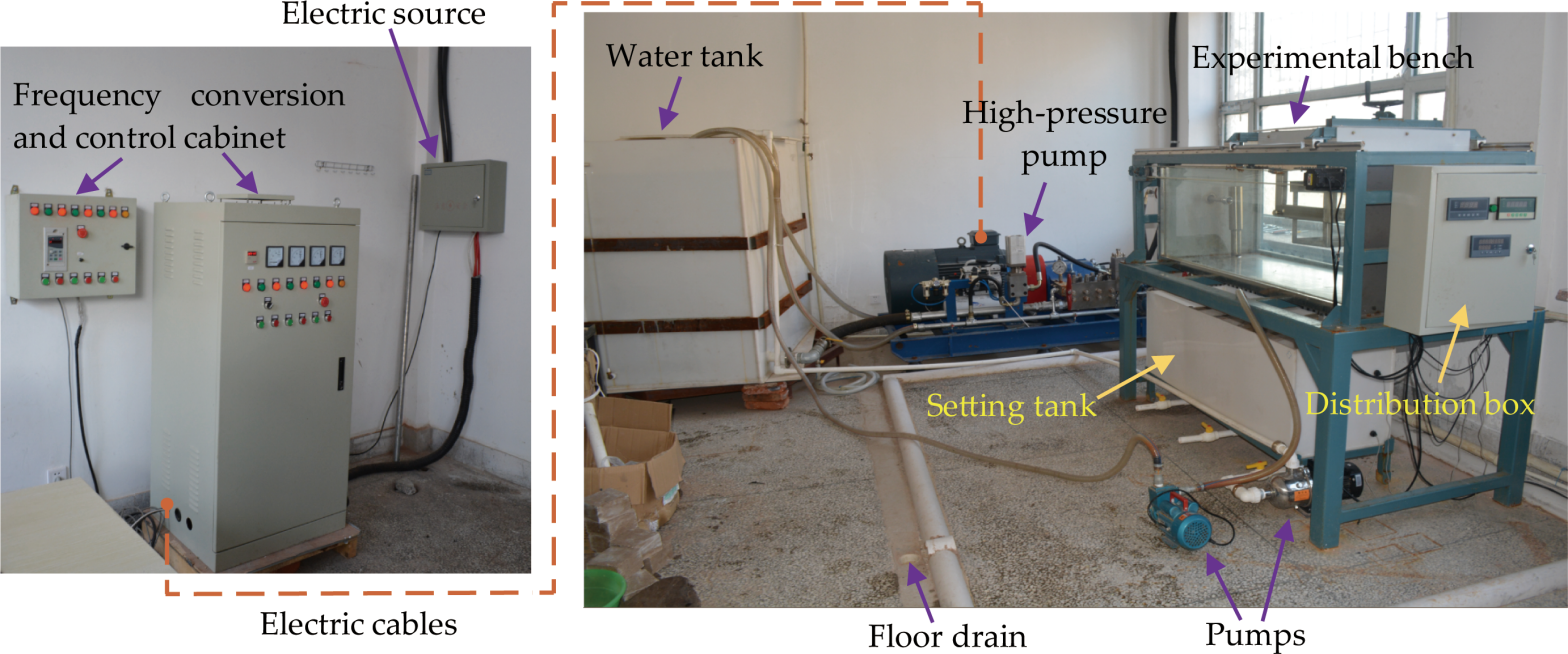
The photograph of the self-developed multifunctional experimental device [18].

**Fig 8:**
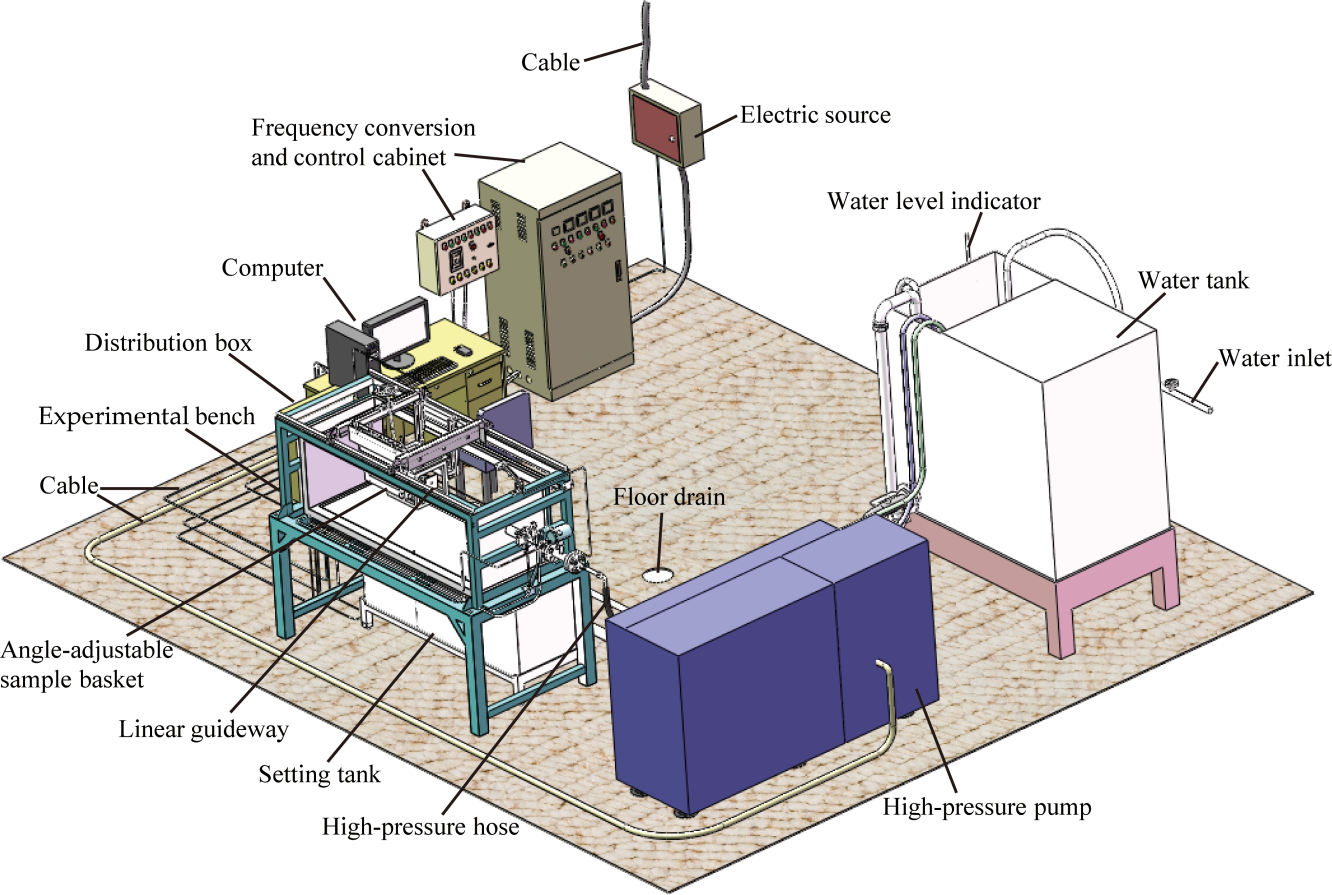
The 3D model of the self-developed multifunctional experimental device.

To compare the jet performances of the three different nozzles and propose the parameters of the borehole hydraulic mining for oil shale through laboratory experiments, the multifunctional experimental device was independently developed by the authors. Fig 7 shows the photograph of the self-developed multifunctional experimental device. Fig 8 shows the 3D model of the self-developed multifunctional experimental device. This experimental device consists of five components. The first is the experimental bench (Ⅰ), which is the frame structure of the experimental device. The second is the power system of the water jet (Ⅱ), its main function is to power the power source of the water jet and the executive elements. The third is the control and detection system (Ⅲ), it allows to change and control the working conditions of the water jet (such as injection pressure, flow, jet distance, and jet angle), as well as to detect, display, and record the technical parameters of the water jet in real-time (such as injection pressure, flow, and jet striking force). The fourth is the fastening and moving system (Ⅳ), used to fasten the oil shale samples and the target plate, which alternately moves backward and forward as well as rotates at the jet angle between 0° and 90°. The fifth is the fluid circulation system (Ⅴ), it ensures water supply, filtration, closed cycle and drain. Additional details about the testing apparatus and procedure can be found in the references [14,18,31]. It is necessary to note that the rated pressure of the high-pressure pump is 15 MPa, and the length of the experimental bench is limited, the total available length for experiment is 984.99 mm. In addition, as discussed above, the total lengths of the different nozzles are also different. Therefore, when different nozzles are connected to the high-pressure pipe, the maximum jet distances that can be adjusted are also different. When the straight cone nozzle is installed, the maximum jet distance is 930 mm. Moreover, when the organ pipe nozzle is installed, the maximum jet distance is 923.59 mm. Furthermore, when the self-excited oscillation pulsed jet nozzle is installed, the maximum jet distance is 841 mm.

**Fig 9:**
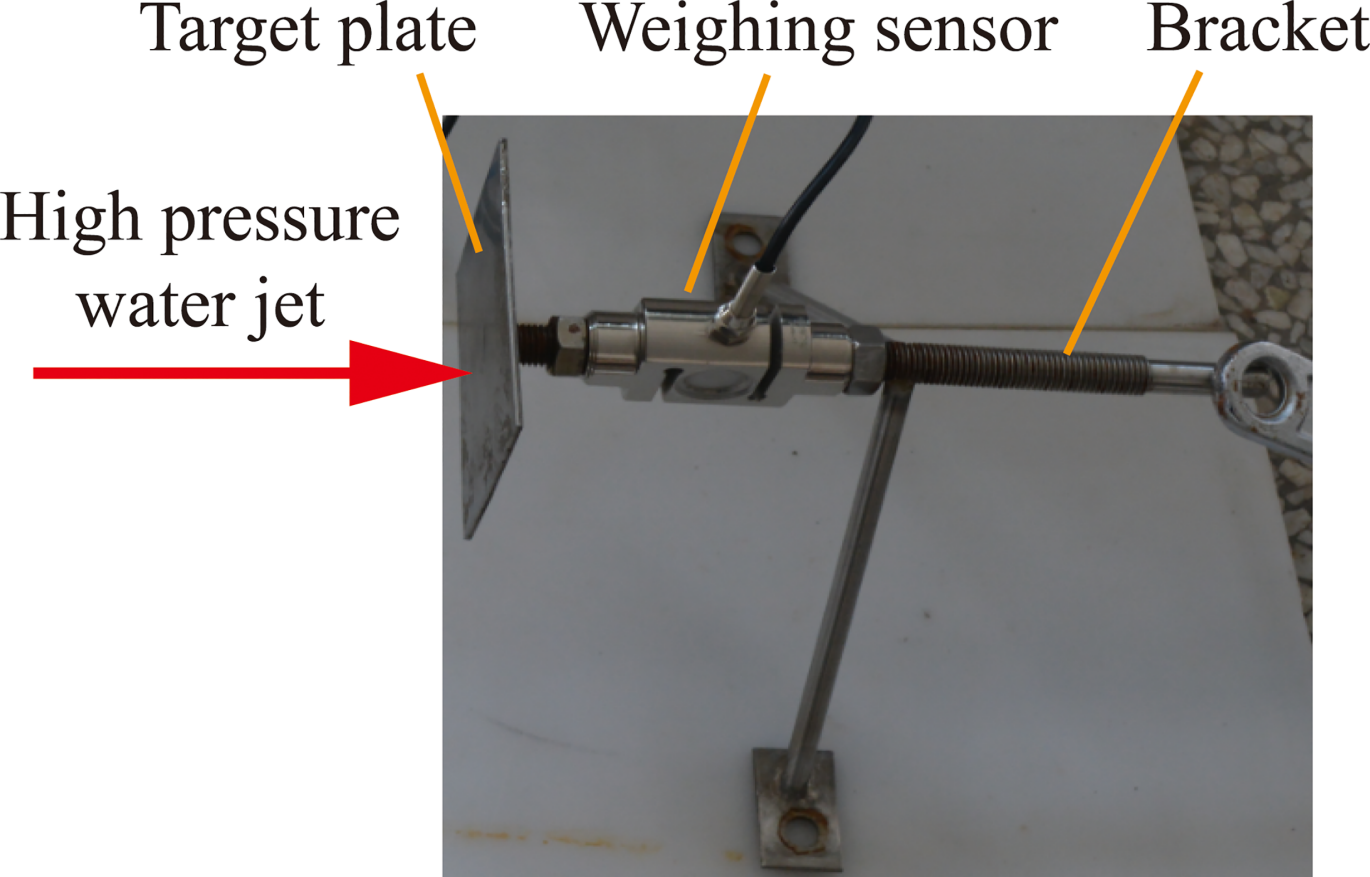
Detection mechanism of the water jet’s striking force [18].

Fig 9 shows the detection mechanism of the water jet’s striking force, which is fixed in the angle-adjustable sample basket. It mainly consists of a target plate, weighing sensor, and bracket. When a high-pressure water jet beam impacts the target plate, the weighing sensor can detect the real-time value of the water jet’s striking force. It is necessary to note that because the weighing sensor cannot operate underwater, the detection mechanism of the water jet’s striking force can only be used to measure the real-time striking force value of the non-submerged water jet. That means it can only measure the real-time striking force of the direct water jet which is produced by the straight cone nozzle, and the pulsed water jet which is produced by the self-excited oscillation pulsed jet nozzle.

### Experimental research on the water jet performances of the jet devices

**Fig 10:**
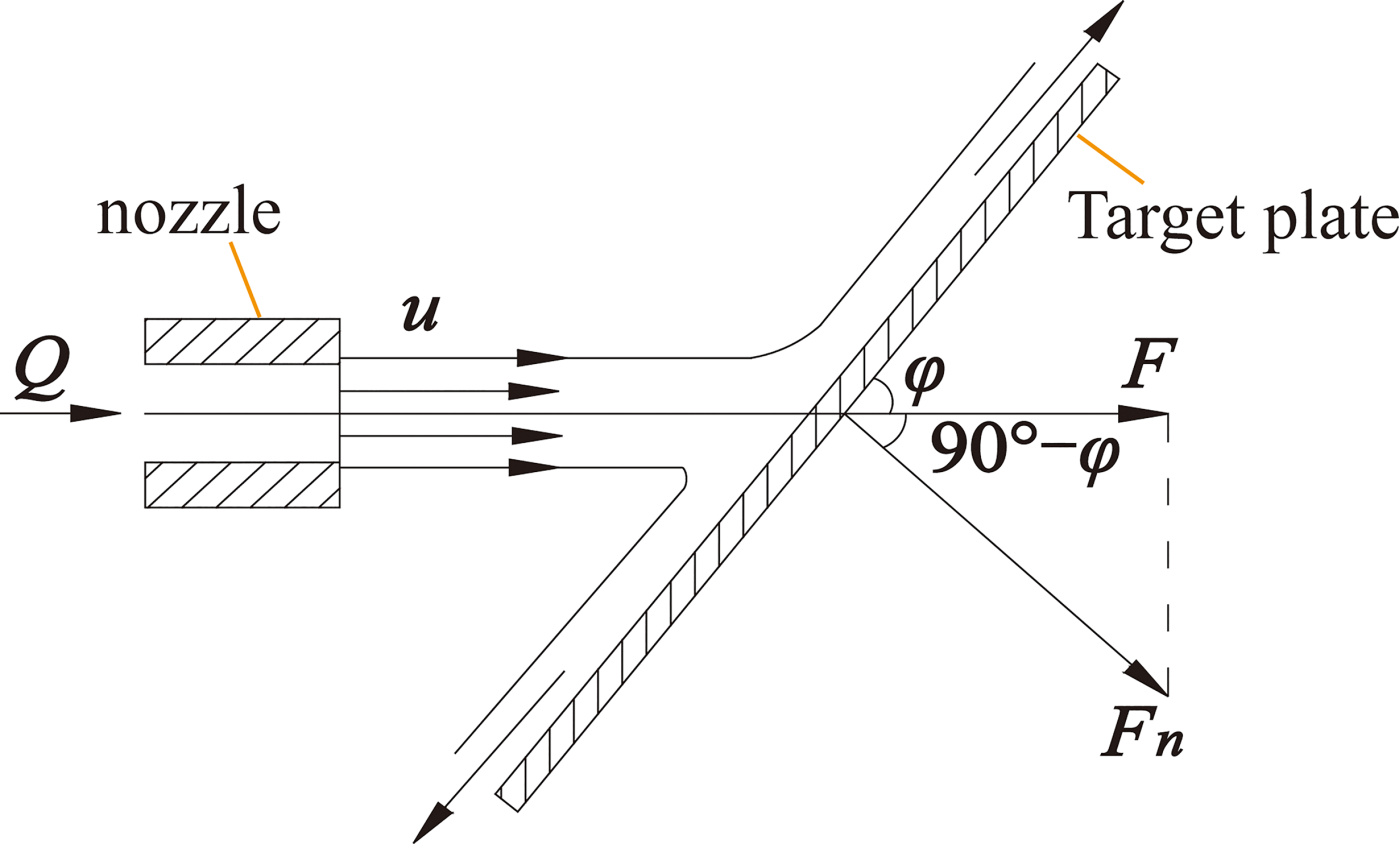
Striking force analysis model of the high-pressure water jet impacts on the target plate [18].

As mentioned above, the water jet performances of the different nozzles are evaluated based on the striking forces of high-pressure water jets under the same experimental conditions. Fig 10 shows the striking force analysis model of the high-pressure water jet impacts on the target plate.

As mentioned in the references [14,18,21], the jet angle of the high-pressure water jet (90°-*φ*) is computed as the angle between the water jet direction and the vertical axis of the target plate. The high-pressure water jet’s striking force on the target plate complies with the following equation:

$$F=F_{n}\cdot \sin\varphi =\rho \cdot Q\cdot u\cdot \sin^{2}\varphi$$ (21)

Where, *F* is the high-pressure water jet’s striking force on the target plate, *F*_*n*_ is the component force of *F* in the vertical direction of the target plate, *φ* is the angle between the high-pressure water jet and the target plate, *ρ* is the density of water, *Q* is the flow of water, and *u* is the velocity of the high-pressure water jet.

When the angle between the high-pressure water jet and the target plate (*φ*) is 90°, the high-pressure water jet’s striking force on the target plate (*F*) can reach the maximum value, and the corresponding jet angle of the high-pressure water jet (90°-*φ*) is 0°. Therefore, the high-pressure water jet cutting or breaking depth of the material is inversely proportional to the jet angle. Under certain operating conditions, the maximum cutting or breaking depth can be obtained when the high-pressure water jet impacts the surface of the material perpendicularly and while the corresponding jet angle is 0° [14,18,21]. Therefore, the jet angle used in all the experiments in this study was 0°.

**Fig 11:**
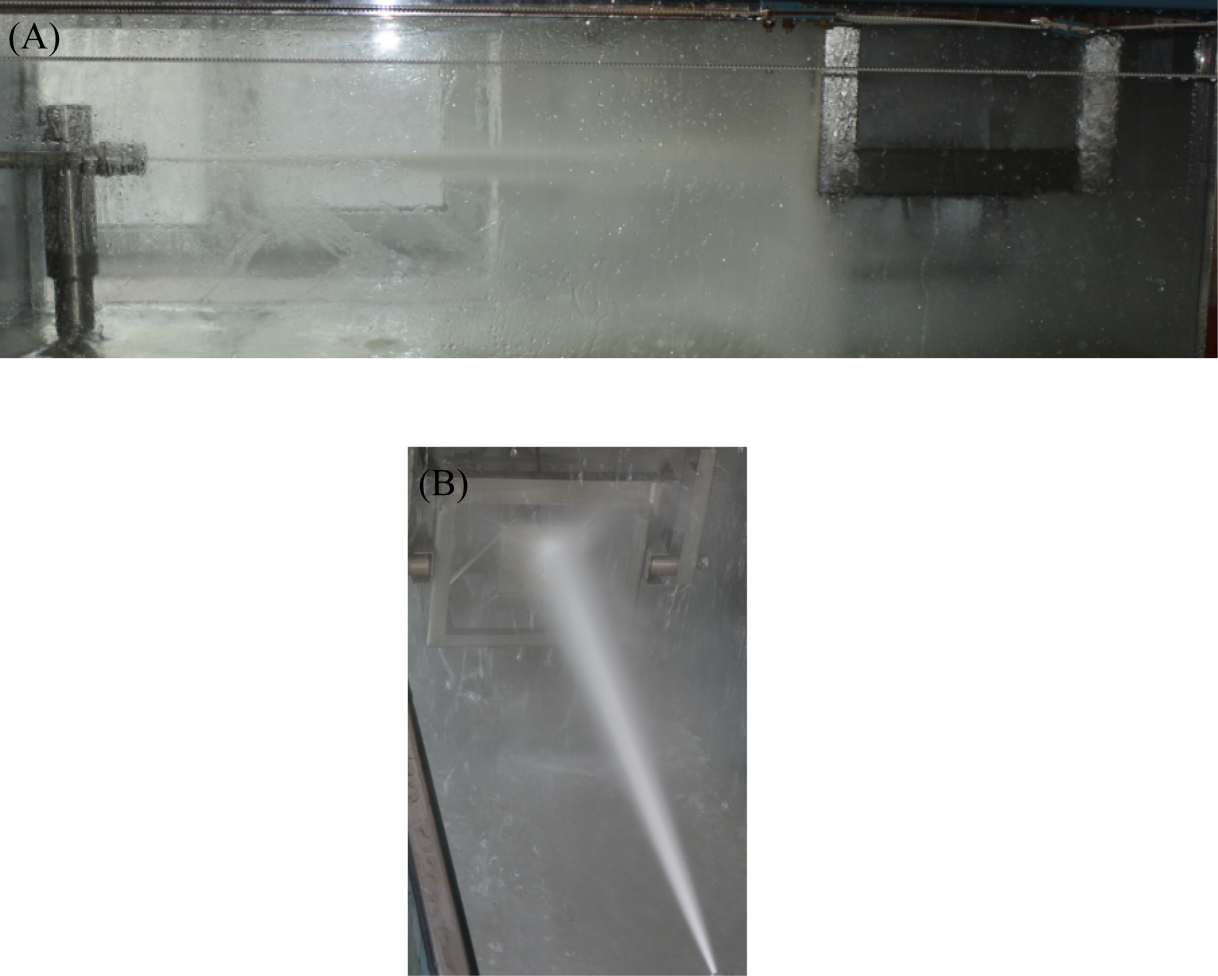
Experimental process of the water jet’s striking force testing. (A) Front view of the experimental process of the water jet’s striking force testing. (B) Vertical view of the experimental process of the water jet’s striking force testing.

As mentioned above, the detection mechanism of the water jet’s striking force can only be used to measure the real-time striking force value of the non-submerged water jet. To compare the operating conditions of these three nozzles, only the organ pipe nozzle is used underwater, and the other two are used above water. Therefore, the water jet striking forces of the straight cone nozzle and the self-excited oscillation pulsed jet nozzle can be measured by the detection mechanism of the water jet’s striking force. Fig 11 shows the experimental process of the water jet’s striking force testing.

**Fig 12:**
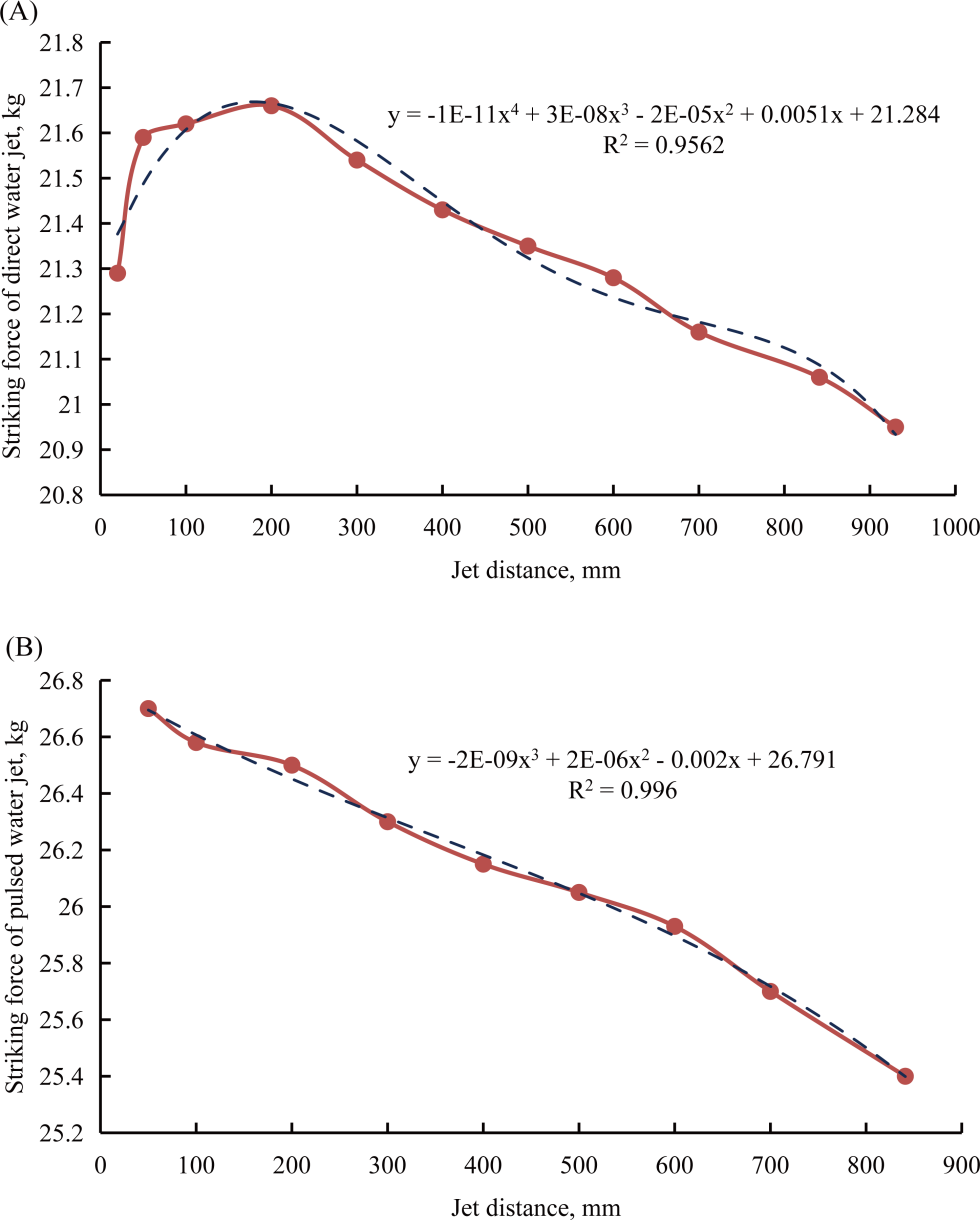
Relationship between the jet distance and the striking force. (A) Relationship between the jet distance and the striking force with the straight cone nozzle. (B) Relationship between the jet distance and the striking force with the self-excited oscillation pulsed jet nozzle.

**Fig 13:**
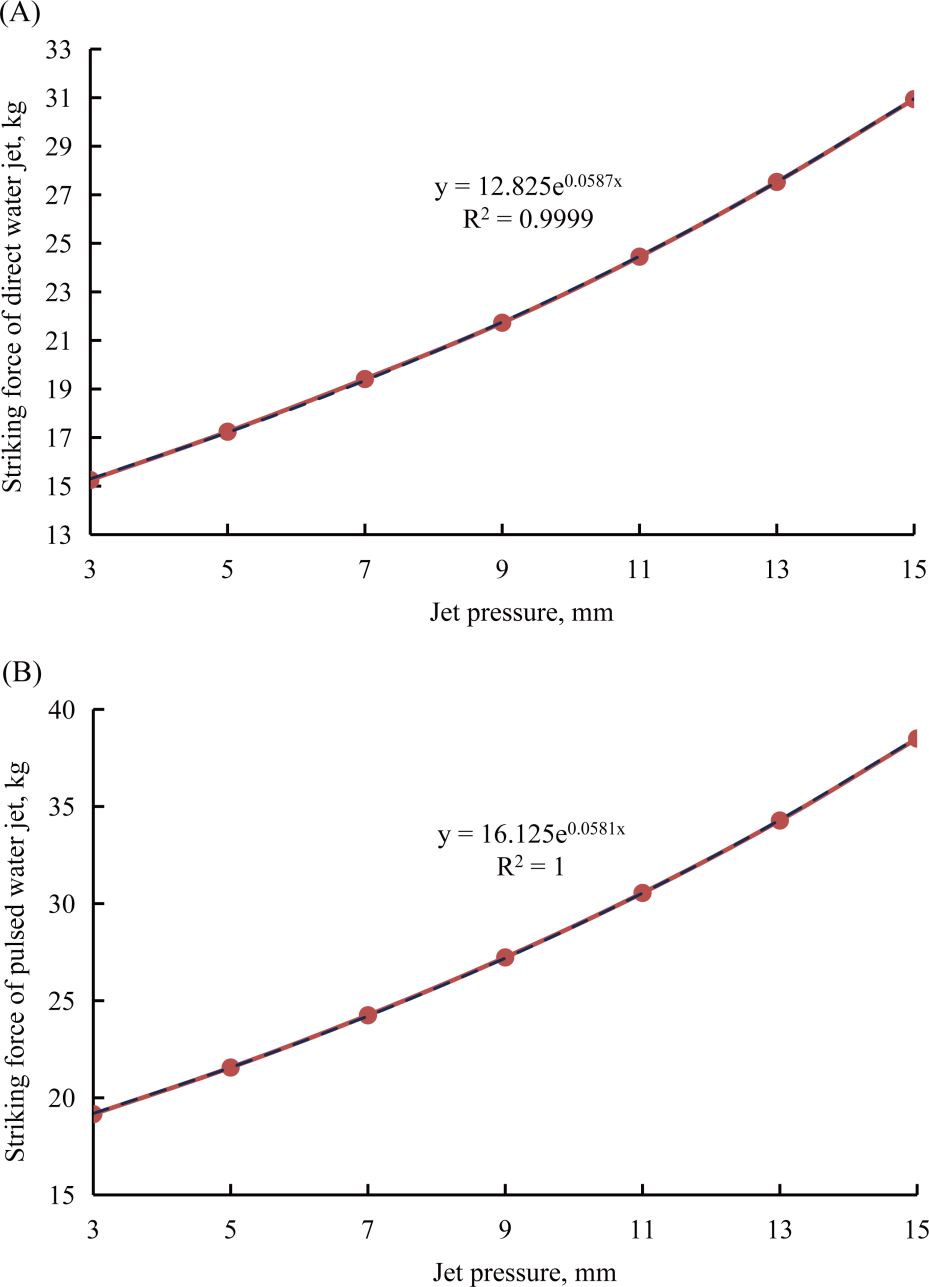
Relationship between the jet pressure and the striking force. (A) Relationship between the jet pressure and the striking force with the straight cone nozzle. (B) Relationship between the jet distance and the striking force with the self-excited oscillation pulsed jet nozzle.

In order to properly compare the striking forces that the high-pressure water jets produced by straight cone nozzle and the self-excited oscillation pulsed jet nozzle, there are two experimental schemes. First, to keep the jet pressure constant and adjust the jet distance. Second, to keep the jet distance constant and adjust jet pressure. In the process of striking force testing of pulsed water jet is produced by the self-excited oscillation pulsed jet nozzle, when the jet distance is 20 mm, the angle-adjustable sample basket cannot be fixed, therefore moving backwards under the impact of the pulsed water jet, so as to change the jet distance easily. For this reason, it is very difficult to test the striking force of the pulsed water jet at the position of 20 mm jet distance. As mentioned above, to better compare the striking force of the water jet of these two nozzles, the experimental conditions include that the jet angle, jet pressure, and jet distance should be uniform. Table 7 shows the details of the experimental schemes and values of the high-pressure water jets’ striking forces testing. Fig 12 shows the relationship between the jet distance and the striking force under the experimental conditions of the jet angle is 0° and the jet pressure is 8 MPa with these two different nozzles. Fig 13 shows the relationship between the jet pressure and the striking force under the experimental conditions of the jet angle is 0° and the jet distance is 841 mm with these two different nozzles.

**Table 7:** Experimental schemes and values of the high-pressure water jets’ striking forces testing.

Experimental Conditions	Straight Cone Nozzle	Self-excited Oscillation Pulsed Jet Nozzle	Straight Cone Nozzle	Self-excited Oscillation Pulsed Jet Nozzle	Experimental Types
	Values of experimental conditions		Values of water jets’ striking force testing		
Jet angle	0 (°)		N/A		Keep the jet pressure constant and adjust the jet distance
Jet pressure	8 (MPa)		N/A		
Jet distance	20 (mm)		21.29 (kg)	N/A	
	50 (mm)		21.59 (kg)	26.70 (kg)	
	100 (mm)		21.62 (kg)	26.58 (kg)	
	200 (mm)		21.66 (kg)	26.50 (kg)	
	300 (mm)		21.54 (kg)	26.30 (kg)	
	400 (mm)		21. 43 (kg)	26.15 (kg)	
	500 (mm)		21.35 (kg)	26.05(kg)	
	600 (mm)		21.28 (kg)	25.93 (kg)	
	700 (mm)		21.16 (kg)	25.70 (kg)	
	841 (mm)		21.06 (kg)	25.40 (kg)	
	930 (mm)		20.95 (kg)	N/A	
Jet distance	841 (mm)		N/A		Keep the jet distance constant and adjust jet pressure
Jet pressure	3 (MPa)		15.25 (kg)	19.16 (kg)	
	5 (MPa)		17.24 (kg)	21.56 (kg)	
	7 (MPa)		19.41 (kg)	24.25 (kg)	
	9 (MPa)		21.73 (kg)	27.23 (kg)	
	11 (MPa)		24.45 (kg)	30.55 (kg)	
	13 (MPa)		27.53 (kg)	34.28 (kg)	
	15 (MPa)		30.94 (kg)	38.50 (kg)	

### Analysis of experimental results of the water jet performances of the jet devices

In Fig 12 and Fig 13, the red curve represents the striking force of the water jet, and the black dotted curve represents the trend curve of the experimental curve of the striking force. Moreover, the equation next to the trend curve is the equation for describing the trend curve. In addition, per the principle of regression analysis, the *R*^2^ is a number between 0 and 1. When the *R*^2^ gets closer to 1, the trend curve is closer to the experimental curve of the striking force. Furthermore, the equation can describe the trend curve very well. In the meantime, the equations also can describe the experimental curve of the striking force very well. There are seven conclusions that can be obtained from Fig 12 and Fig 13:

First, in Fig 12A, the *R*^2^ is 0.9562, so the experimental curve of the striking force of the direct water jet is produced by the straight cone nozzle matches the trend curve well. When the jet distance is less than 200 mm, the striking force of the direct water jet increases with the increases of the jet distance. However, when the jet distance is greater than 200 mm, the striking force of the direct water jet decreases with the increases of the jet distance. That means that there exists an optimal jet distance. In this study, the optimal jet distance is 200 mm, it is fifty times the diameter of the straight cone nozzle outlet (*d* = 4 mm). The optimal jet distance of the best designed straight cone nozzle under the excellent working conditions is about 50 to 100 times the diameter of its outlet [22]. The experimental results are consistent with this observation. In addition, the trend curve and the experimental curve of the striking force can be described as a quartic polynomial equation as follows:

$$y=-1\times 10^{-11}\cdot x^{4}+3\times 10^{-8}\cdot x^{3}-2\times 10^{-5}\cdot x^{2}+5.1\times 10^{-3}\cdot x+21.284$$ (22)

Where, *x* is the jet distance, *y* is the striking force of direct water jet.

Second, in Fig 12B, the *R*^2^ is 0.996, so the experimental curve of the striking force of the pulsed water jet is produced by the self-excited oscillation pulsed jet nozzle matches the trend curve very well. When the jet distance is greater than or equal to 50 mm, the striking force of the pulsed water jet decreases with the increases of the jet distance. In addition, the trend curve and the experimental curve of the striking force can be described as a cubic polynomial equation as follows:

$$y=-2\times 10^{-9}\cdot x^{3}+2\times 10^{-6}\cdot x^{2}-2\times 10^{-3}\cdot x+26.791$$ (23)

Where, *x* is the jet distance, *y* is the striking force of pulsed water jet.

Third, to compare Fig 12A and 12B is not difficult to find that the striking forces of the direct water jet and the pulsed water jet are respectively produced by the straight cone nozzle and the self-excited oscillation pulsed jet nozzle are different. Under the same experimental conditions, this means the jet angle is 0°, the jet pressure is 8 MPa, the inlet diameter of these two nozzles is 19 mm. The striking force of the pulsed water jet that is produced by the self-excited oscillation pulsed jet nozzle is always greater than the striking force of the direct water jet that is produced by the straight cone nozzle at the same jet distance.

Fourth, in Fig 13A, the *R*^2^ is 0.9999, so the experimental curve of the striking force of the direct water jet is produced by the straight cone nozzle can match the trend curve very well. The striking force of the direct water jet increases with the increases of the jet pressure. In addition, the trend curve and the experimental curve of the striking force can be described as an exponential equation as follows:

$$y=12.825\cdot e^{0.0587x}$$ (24)

Where, *x* is the jet pressure, *y* is the striking force of direct water jet.

Fifth, in Fig 13B, the *R*^2^ is 1, so the experimental curve of the striking force of the pulsed water jet is produced by the self-excited oscillation pulsed jet nozzle fits the trend curve perfectly. The striking force of the pulsed water jet increases with the increases of the jet pressure. In addition, the trend curve and the experimental curve of the striking force can be described as an exponential equation as follows:

$$y=16.125\cdot e^{0.0581x}$$ (25)

Where, *x* is the jet pressure, *y* is the striking force of pulsed water jet.

Sixth, to compare Fig 13A and 13B is not difficult to find that the striking forces of the direct water jet and the pulsed water jet are respectively produced by the straight cone nozzle and the self-excited oscillation pulsed jet nozzle are different. Under the same experimental conditions, that means the jet angle is 0°, the jet distance is 841 mm, the inlet diameter of these two nozzles is 19 mm, but the striking force of the pulsed water jet is produced by the self-excited oscillation pulsed jet nozzle is always greater than the striking force of the direct water jet is produced by the straight cone nozzle under the same jet pressure.

As discussed above, the striking force of the pulsed water jet which is produced by the self-excited oscillation pulsed jet nozzle is always greater than the direct water jet which is produced by the straight cone nozzle under the same experimental conditions. It also means the water jet performance of the self- excited oscillation pulsed jet nozzle is always better than that of the straight cone nozzle.

### Experimental study of breaking oil shale with the jet devices

In this study, the oil shale samples were all taken from Sunjia Village, Huadian City, Jilin Province, China. It is necessary to note that the oil shale has a clear stratification (schistosity) characteristic, and it will be affected by weathering in the process of transporting from the field site to the laboratory. Therefore, data is listed in Table 8 which is based on the cores drilled in the field in order to obtain the real mechanical properties of the oil shale in Huadian. Its geographical coordinate is (42°59′19″ N, 126°50′09″ E). The buried depth of oil shale seam is about 750m. Our research group is based on the Standard for Test Methods of Engineering Rock Mass (GB/T 50266–99) which is the national standard of the People’s Republic of China, to test the fresh oil shale samples’ physical and mechanical properties [32]. Table 8 shows the mechanical properties of these oil shale samples.

**Table 8:** Mechanical properties of the oil shale in Huadian.

Mechanical Properties	Compressive Strength, MPa	Tensile Strength, MPa	Elastic Modulus, MPa	Poisson Ratio	Internal Friction angle, °	Cohesive Force, MPa
Value range	9 to 19	0.8 to 1.5	216 to 386	0.27 to 0.35	15 to 33	0.7 to 1.5

The parameters of the borehole hydraulic mining technology mainly include jet pressure (jet flow), jet angle, jet distance, and the relationship between the jet direction and the bedding direction of oil shale. The external environment of the high-pressure water jet also has a great influence on the broken oil shale. The parameters that are mentioned above will have a great impact on the efficiency of water jet broken oil shale and the scheme of borehole hydraulic mining. Moreover, the time of the high-pressure water jet is continuously ejected to the surface of the oil shale until the oil shale is broken is recorded, which can be used as an important reference index for measuring the oil shale breaking efficiency of the jet devices. In addition, considering of the schistosity of oil shale and its obvious bedding development, it is necessary to note that the experiment should be divided into two schemes under the same experimental conditions: First, the high-pressure water jet direction is parallel to the bedding direction of oil shale. Second, the high-pressure water jet direction is perpendicular to the bedding direction of oil shale. The first experiment of breaking oil shale consists of using the direct water jet which is produced by the straight cone nozzle. The details of the experimental conditions are listed in Table 9.

**Table 9:** Experimental conditions of breaking oil shale with the straight cone nozzle.

Serial Number	Experimental Conditions	Values
1	Jet pressure	15 (MPa)
2	Jet flow	6.79 (m^3^/h)
3	Jet angle	0 (°)
4	Jet distance	200 (mm)
5	External environment	Air
6	Type of water jet	Non-submerged direct water jet
7	Jet direction	(1) The direct water jet direction is parallel to the bedding direction of oil shale. (2) The direct water jet direction is perpendicular to the bedding direction of oil shale.

**Fig 14:**
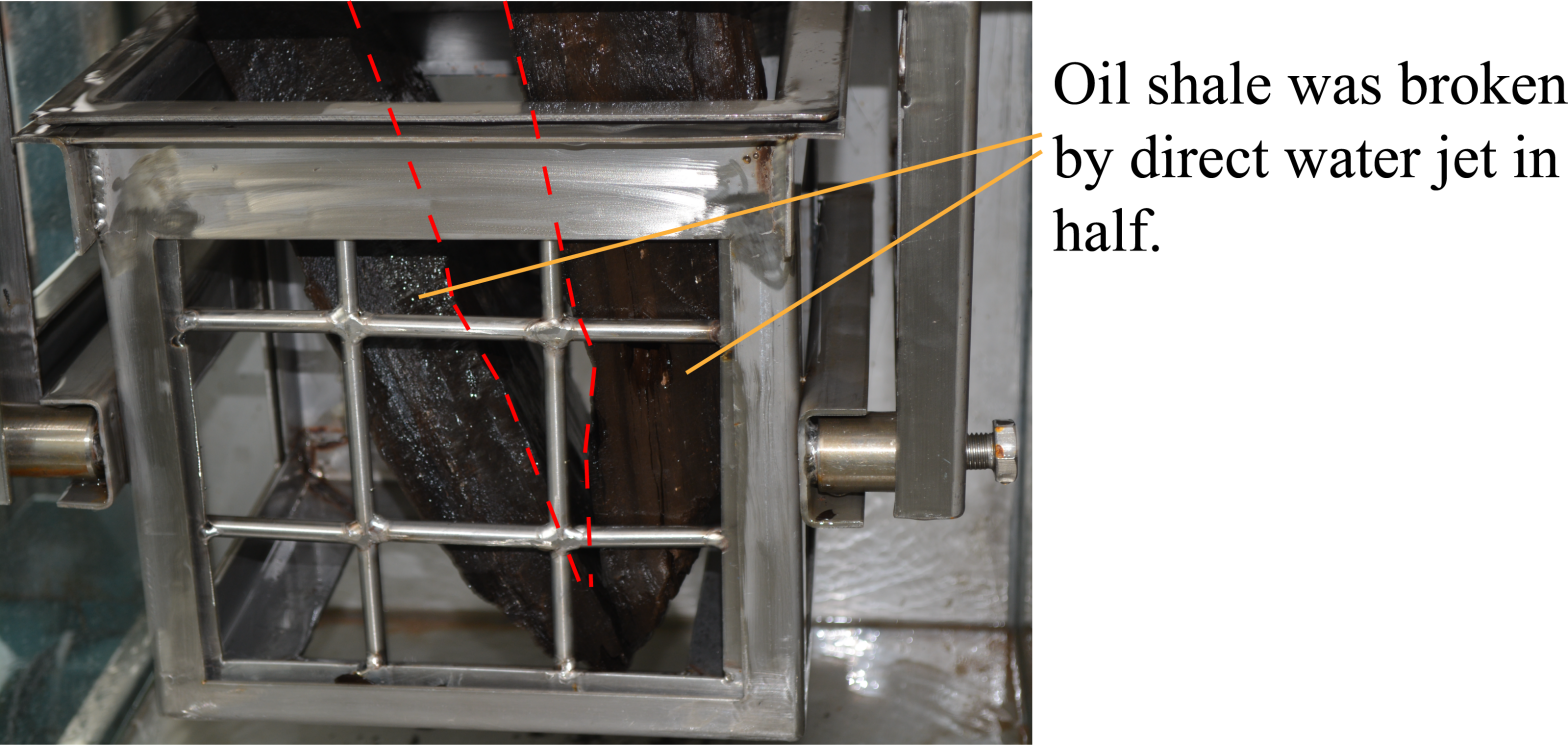
Broken oil shale sample by the direct water jet which is parallel to the bedding of oil shale.

**Fig 15:**
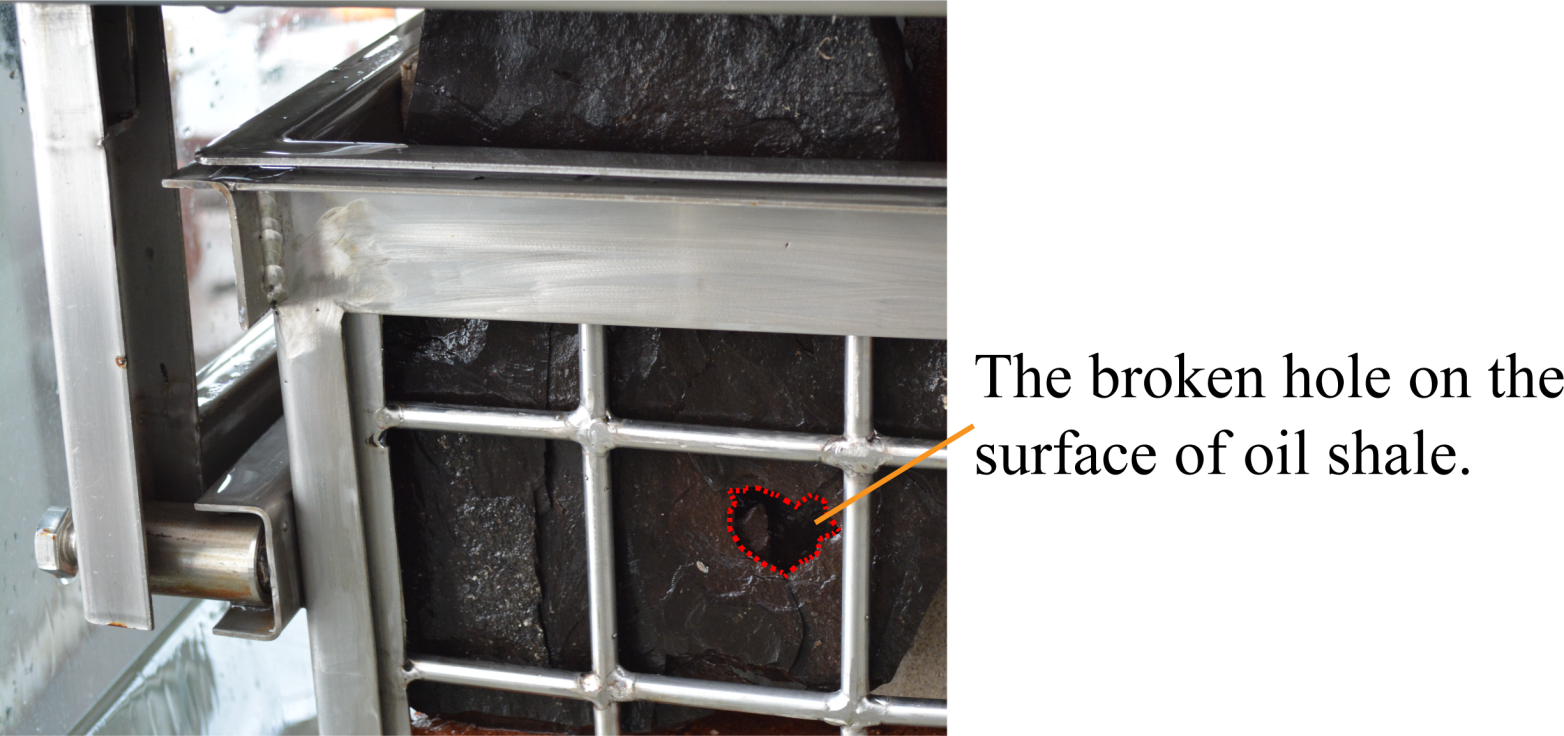
Broken oil shale sample by the direct water jet which is perpendicular to the bedding of oil shale.

When the direct water jet direction is parallel to the bedding direction of oil shale, the entire experiment lasted 10 seconds and the oil shale was broken in half along its bedding. Fig 14 shows the broken oil shale sample by the direct water jet which is parallel to the bedding of oil shale. Moreover, to use one of the oil shale that has been broken in half as the experimental oil shale sample to do the next experiment. When the direct water jet direction is perpendicular to the bedding direction of oil shale, the entire experiment lasted 90 seconds and only a broken hole appeared on the surface of the oil shale sample, but the entire oil shale is not completely broken, as shown in Fig 15. The size of the broken hole is about 53 mm × 65 mm, and its depth is 5 mm. More details of the direct water jet which is produced by the straight cone nozzle broken oil shale can be learned from reference [14,18].

The second experiment of breaking oil shale is used the cavitating water jet which is produced by the self-resonating cavitating jet nozzle. The details of the experimental conditions are listed in Table 10.

**Table 10:** Experimental conditions of breaking oil shale with the self-resonating cavitating jet nozzle.

Serial Number	Experimental Conditions	Values
1	Jet pressure	15 (MPa)
2	Jet flow	2.87 (m^3^/h)
3	Jet angle	0 (°)
4	Jet distance	400 (mm), 100 (mm), 200 (mm), 150 (mm), 30 (mm)
5	External environment	Water
6	Type of water jet	Submerged cavitating water jet
7	Jet direction	(1) The cavitating water jet direction is parallel to the bedding direction of oil shale. (2) The cavitating water jet direction is perpendicular to the bedding direction of oil shale.

**Fig 16:**
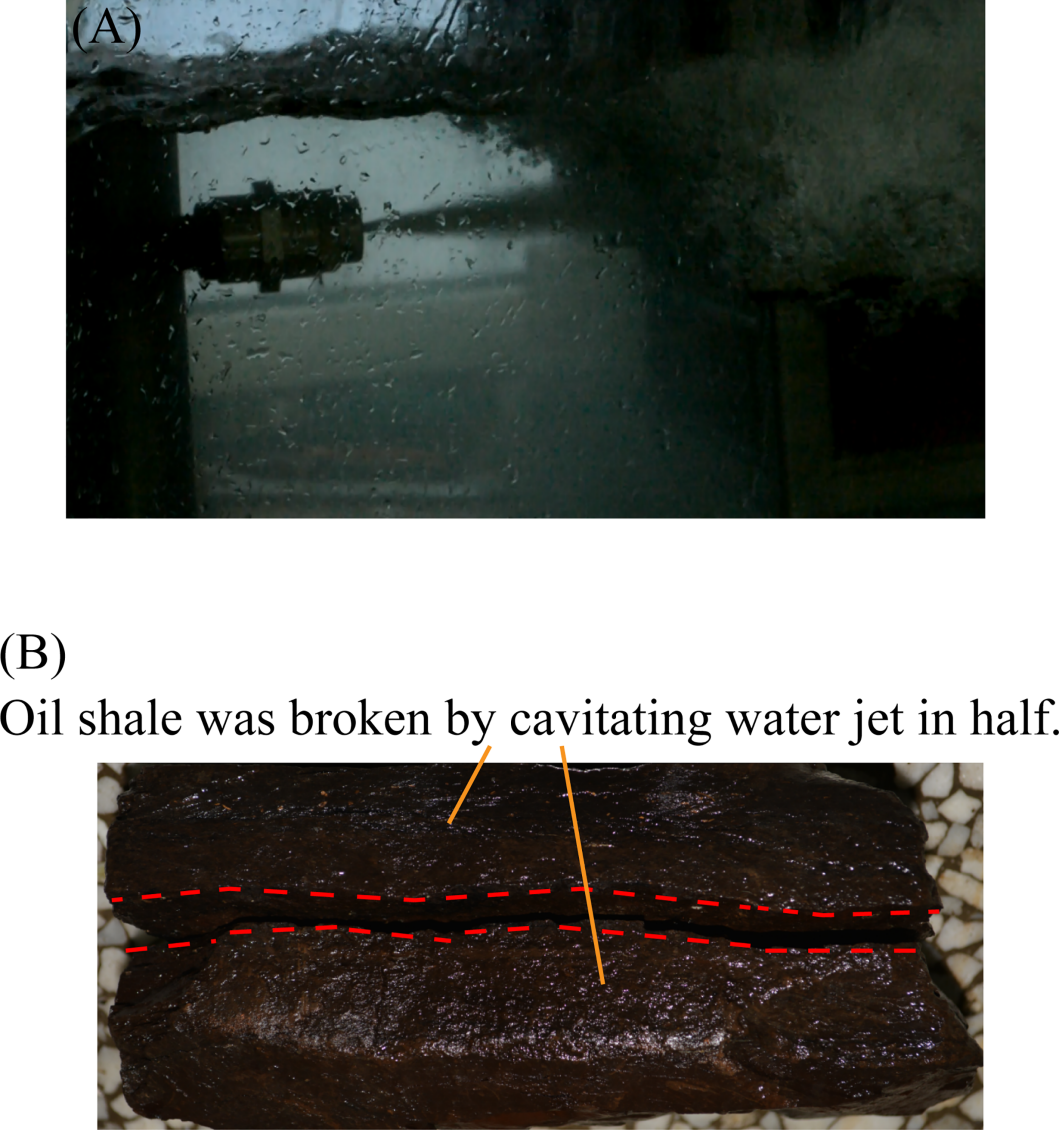
First broken oil shale sample by the cavitating water jet which is parallel to the bedding of oil shale. (A) Cavitating water jet is produced by the self-resonating cavitating jet nozzle underwater. (B) First broken oil shale sample by the cavitating water jet.

**Fig 17:**
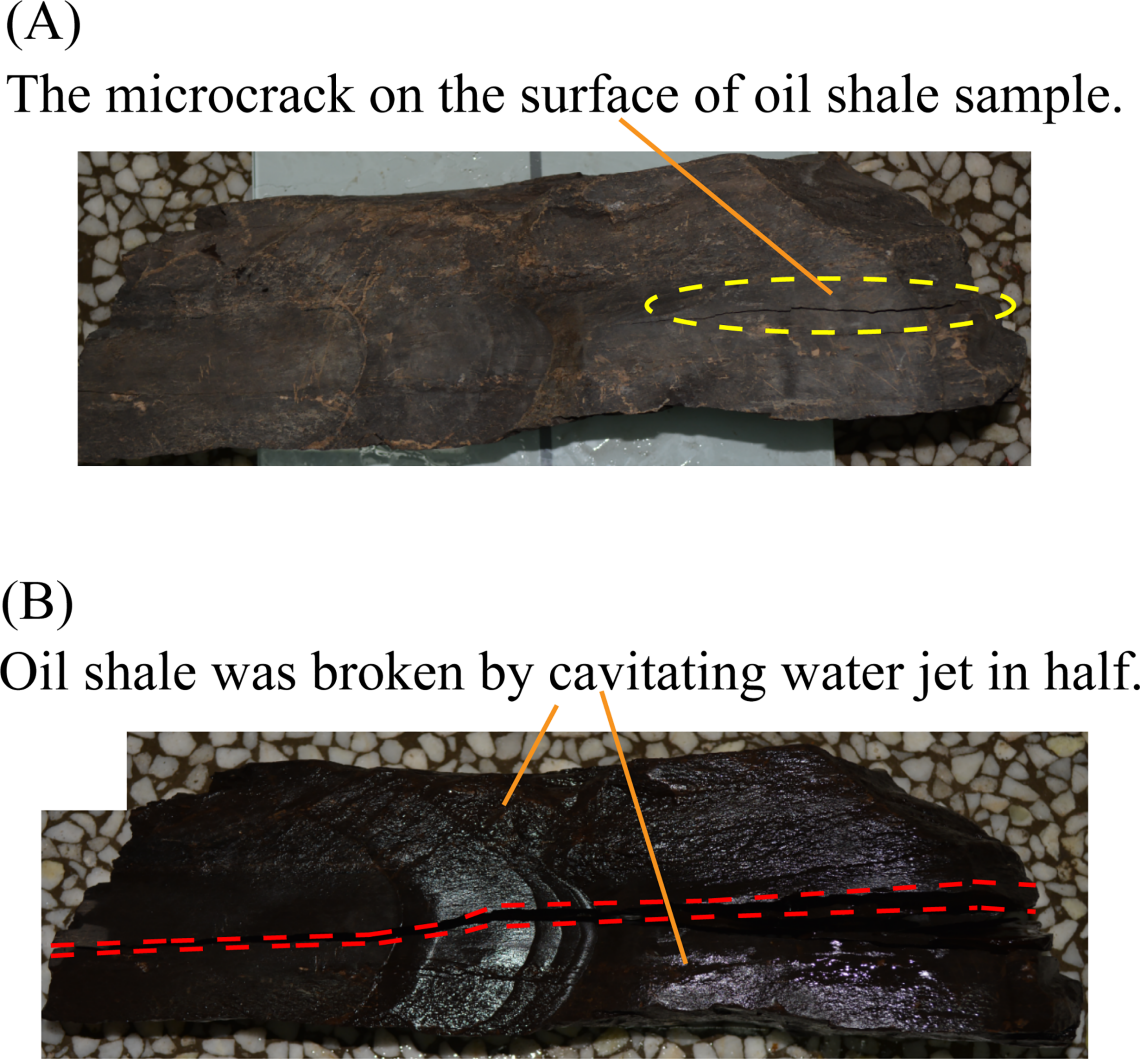
Second broken oil shale sample with a surface microcrack by the cavitating water jet which is parallel to the bedding of oil shale. (A) Oil shale sample with a surface microcrack. (B) Second broken oil shale sample by the cavitating water jet.

The relatively complete and dense oil shale is first used as an experimental sample. When the cavitating water jet direction is parallel to the bedding direction of oil shale, and the jet distance is 400 mm, the entire experiment lasted 120 seconds, but the oil shale remained intact. Next, the jet distance was adjusted to 100 mm, the entire experiment only lasted 12 seconds, the oil shale was broken in half along its bedding. Moreover, the oil shale with surface microcrack was used as an experimental sample. When the cavitating water jet direction was parallel to the bedding direction of oil shale, and the jet distance is 200 mm, the entire experiment lasted 60 seconds, but the oil shale is intact. Then, the jet distance was adjusted to 150 mm, the entire experiment only lasted 7 seconds, the oil shale was broken in half along its bedding. Fig 16 shows the first broken oil shale sample by the cavitating water jet which is parallel to the bedding of oil shale. Fig 17 shows the second broken oil shale sample with a surface microcrack by the cavitating water jet which is parallel to the bedding of oil shale.

**Fig 18:**
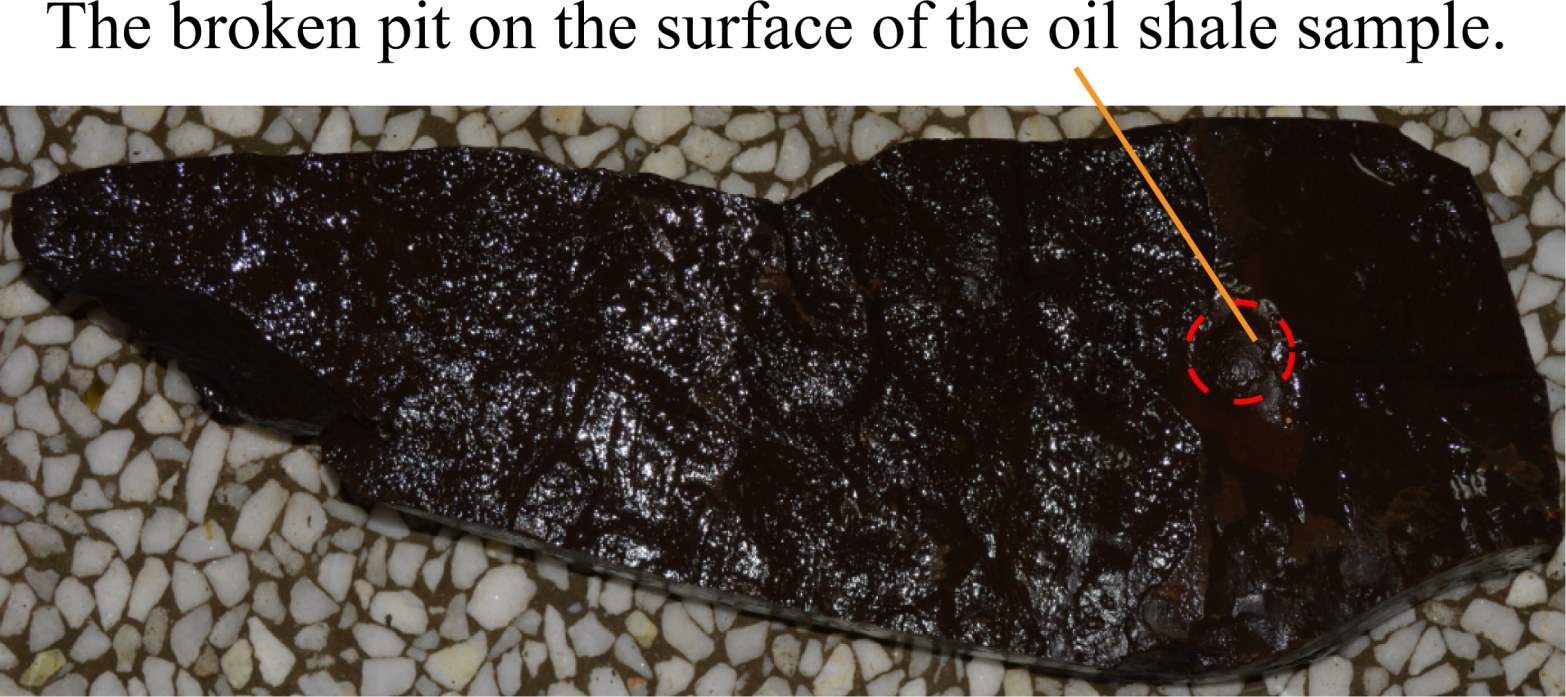
Broken oil shale sample by the cavitating water jet which is perpendicular to the bedding of oil shale.

The oil shale samples that had been broken in half were utilized to do the following experiment. When the cavitating water jet direction was perpendicular to the bedding direction of oil shale, and the jet distance is 150 mm, the entire experiment lasted 50 seconds, but the oil shale remained intact. Also, the jet distance was adjusted to 30 mm, the entire experiment lasted 40 seconds, there was a broken pit appeared on the surface of the oil shale sample, but the entire oil shale was not completely broken, as shown in Fig 18. The size of the broken pit is about 22 mm × 24 mm, and its depth is 6 mm.

The third experiment of breaking oil shale was conducted using the pulsed water jet which is produced by the self-excited oscillation pulsed jet nozzle. The details of the experimental conditions are listed in Table 11.

**Table 11:** Experimental conditions of breaking oil shale with the self-excited oscillation pulsed jet nozzle.

Serial Number	Experimental Conditions	Values
1	Jet pressure	8 (MPa)
2	Jet flow	7.69 (m^3^/h)
3	Jet angle	0 (°)
4	Jet distance	841 (mm), 400 (mm)
5	External environment	Air
6	Type of water jet	Non-submerged pulsed water jet
7	Jet direction	(1) The pulsed water jet direction is parallel to the bedding direction of oil shale. (2) The pulsed water jet direction is perpendicular to the bedding direction of oil shale.

**Fig 19:**
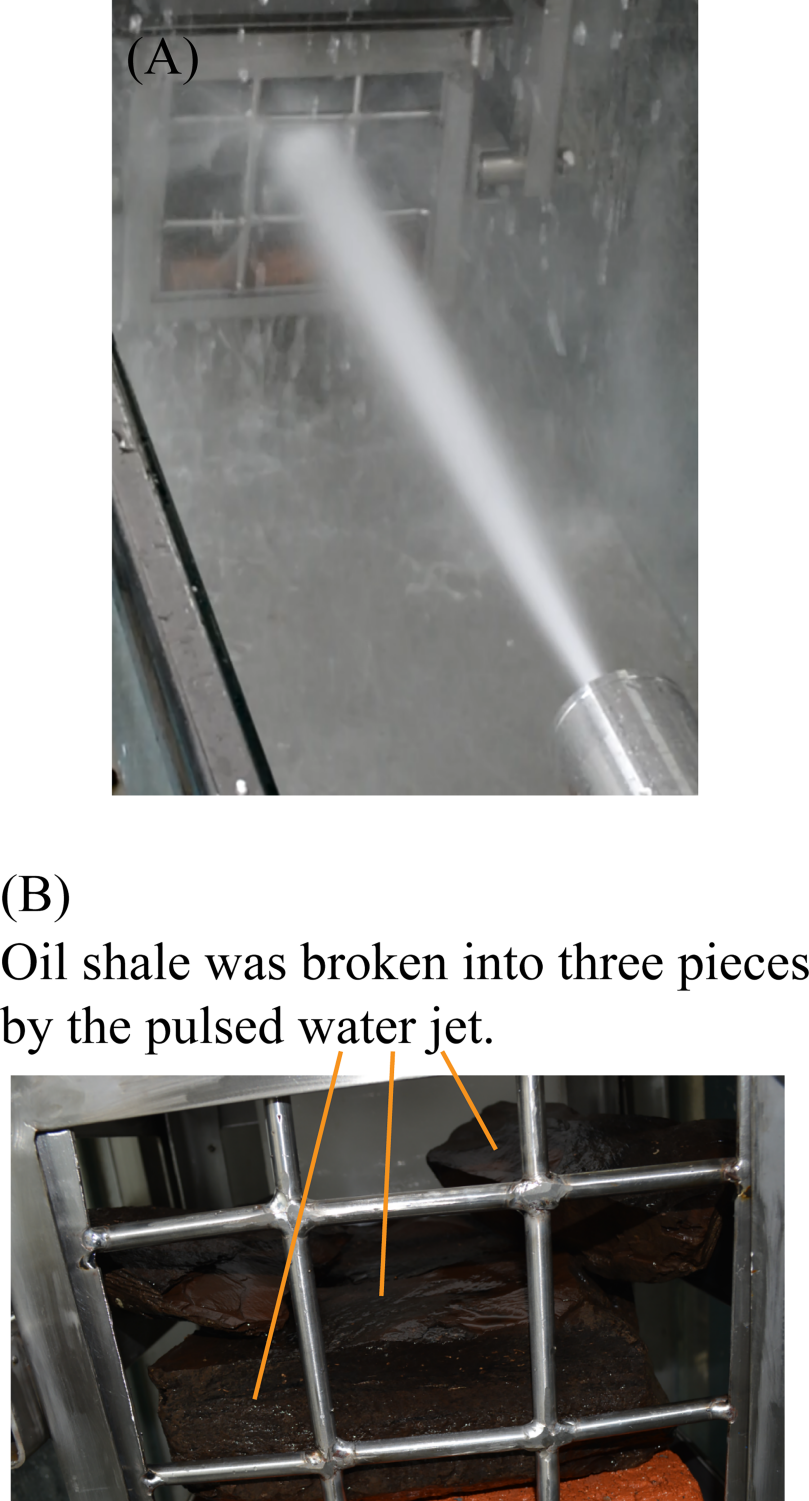
Broken oil shale sample by the pulsed water jet which is parallel to the bedding of oil shale. (A) The pulsed water jet is produced by the self-excited oscillation pulsed jet nozzle. (B) The broken oil shale sample by the pulsed water jet.

When the pulsed water jet direction was parallel to the bedding direction of oil shale, the entire experiment only lasted 9 seconds and the oil shale was broken in half along its bedding. Fig 19 shows the broken oil shale sample by the pulsed water jet which was parallel to the bedding of oil shale.

**Fig 20:**
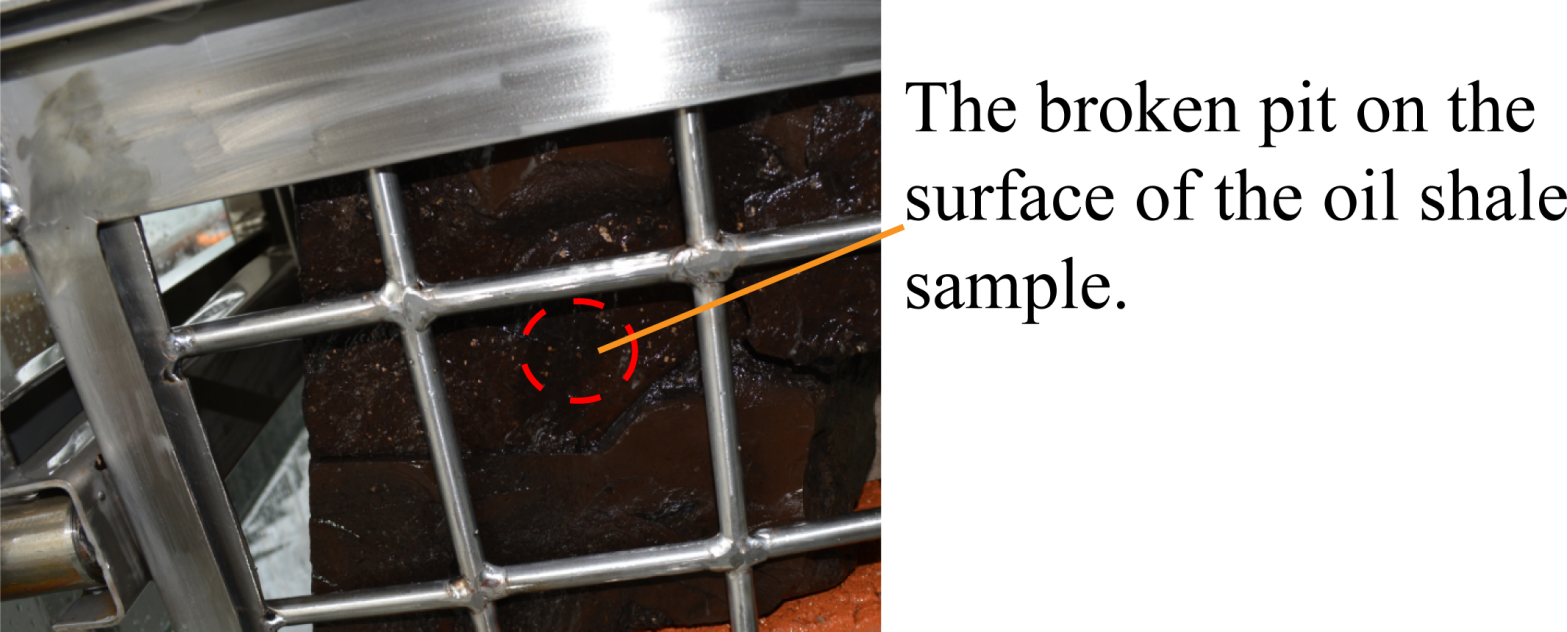
Broken oil shale sample by the pulsed water jet which is perpendicular to the bedding of oil shale.

For the next experiment, the pulsed water jet direction was perpendicular to the bedding direction of oil shale, the entire experiment lasted 60 seconds, but the oil shale remained intact. Then, the jet distance was adjusted to 400 mm, the entire experiment lasted 50 seconds, a broken pit appeared on the surface of the oil shale sample, but the entire oil shale was not completely broken, as shown in Fig 20. The size of the broken pit is about 28 mm × 34 mm, and its depth is 5 mm.

**Fig 21:**
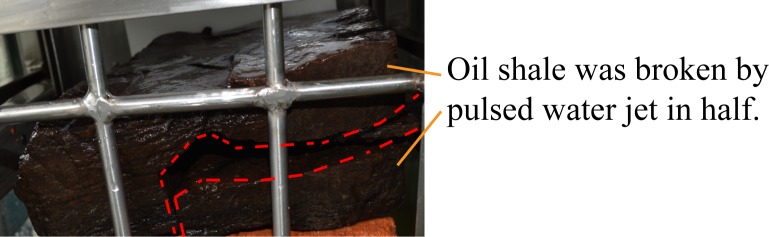
Broken oil shale sample by the pulsed water jet which impacts to the surface microcrack of oil shale.

For the next experiment, the jet distance was adjusted to 841 mm. When the pulsed water jet impacts to the surface crack of oil shale, also the jet direction is perpendicular to the bedding direction of oil shale, the entire experiment only lasted 40 seconds and the oil shale was broken in half along its surface crack, as shown in Fig 21.

### Analysis of experimental results of breaking oil shale with the jet devices

The red dotted lines in Fig 13 to Fig 20 represent the fractured region boundaries on the oil shale sample that were impacted by the different high-pressure water jets. Five conclusions can be obtained from the above experimental results of breaking oil shale:

First, regardless of the water jet type, it is easier to break oil shale when the high-pressure water jets are in the parallel bedding direction than when the water jets are in the vertical bedding direction of the oil shale. This conclusion is mainly determined by the schistosity of oil shale.

Second, the degree of difficulty in breaking oil shale of water jet is related to its own integrity. When there is microcrack or crack in oil shale, it is easier to be broken with water jet.

Third, although the cavitating water jet has a strong ability of cavitation erosion, its effective jet distance to break oil shale is shorter than other two kinds of water jets. The larger energy attenuation of submerged water jet is the main reason for this conclusion. Therefore, the non-submerged water jet is more efficient in breaking oil shale.

Fourth, as the non-submerged water jet, the pulsed water jet is produced by the self-excited oscillation pulsed jet nozzle which has the stronger effect of breaking oil shale under the lower jet pressure. Its effect of breaking oil shale is more significant than the direct water jet which is produced by the straight cone nozzle. The more significant effect of breaking oil shale by the pulsed water jet means that the effective jet distance to break oil shale is longer, the jet time to break oil shale is shorter, and the jet pressure to break oil shale is lower. However, it is necessary to note that when the self-excited oscillation pulsed jet nozzle is used to produce pulsed water jet to break oil shale, it requires a pump to provide more jet flow than the other two types of water jets. Moreover, if the jet pressure continues to be increased, the required jet flow will be greater, so the demand for the capacity of pump will also be higher.

Fifth, in these experiments of breaking oil shale by jet devices, the best effect of breaking oil shale comes from the self-excited oscillation pulsed jet nozzle under the non-submerged condition. Therefore, the best scheme of borehole hydraulic mining for oil shale as follows: Firstly, it is preferred to use the self-excited oscillation pulsed jet nozzle. That also means the optimal jet type is non-submerged pulsed water jet. Secondly, the optimal pulsed water jet direction is parallel to the bedding direction of oil shale. Thirdly, under the preconditions of ensuring the safety and reliability of the hydraulic mining equipment and pipelines connection, the jet pressure and jet flow should be raised as much as possible.

## Discussion

As discussed above, in the process of the borehole hydraulic mining for underground oil shale, the most critical step is using high-pressure water jet which is ejected by the jet device to break the underground oil shale into several small pieces and then peel them off from the parent rock. Moreover, in the case of using only a borehole to implement the borehole hydraulic mining for underground oil shale, the farther the effective water jet distance to break oil shale, the greater the scope of the mining. That means the more oil shale can be mined based on a same borehole. Also, the ability of high-pressure water jet to break the oil shale has crucial influence on the efficiency of the borehole hydraulic mining for underground oil shale. This also means the cost of the borehole hydraulic mining for underground oil shale will be lower. Therefore, to explore a jet device which has excellent water jet performance is very important and meaningful.

Per the results of the theoretical and experimental research, it is concluded that the jet performance of the non-submerged water jet is better than the submerged water jet. It is necessary to note that this conclusion is suitable for the different water jets’ types. Moreover, the pulsed water jet which is produced by the self-excited oscillation pulsed jet nozzle has the best jet performance in these jet devices. Also, all these jet devices have their structural parameters optimized.

It is necessary to note that because the data of the striking force is tested by the weighing sensor, the unit of the water jets’ striking force is kilogram (kg) not Newton (N) in this study. The main reason is that comprehensive considering to make the process of the experiment simple and effective, and the Newton (N) as the force unit can easily convert to kilogram (kg) under the same force area on the surface of target, their relationship is in direct proportion. Furthermore, the water jet beams of these different water jets’ types in this study are all diverging. It means the area of these water jets’ cross section increases with the increase of the jet distance. That also means the force area of the water jet on the surface of target will change as the jet distance changes. It leads to change the unit of the water jet’s striking force from kilogram (kg) to Newton (N) is difficult. Therefore, kilogram (kg) is used as the unit of the water jet’s striking force in this study. In addition, because the weighing sensor cannot operate underwater, the real-time striking force of the cavitating water jet which is produced by the organ pipe nozzle in this study was not measured. In future studies, this problem can be solved by adding a sealed part to make the weighing sensor waterproof or using a waterproof weighing sensor.

Through the above research, the best way to implement the borehole hydraulic mining for underground oil shale is to use the pulsed water jet which is produced by the self-excited oscillation pulsed jet nozzle under the non-submerged condition, and the pulsed water jet should be parallel to the oil shale bedding. In order to achieve this mining condition, the double boreholes or multiple boreholes mining method is presented as the implementation schemes of borehole hydraulic mining for underground oil shale. The double boreholes or multiple boreholes mining method means comprehensive utilization of two or more boreholes for borehole hydraulic mining for underground oil shale. The first step is to drill two or more vertical boreholes through oil shale deposit from ground. Secondly, to use the technology of directional drilling (DD) or horizontal directional drilling (HDD) to make these boreholes connected underground. Third, one of the vertical boreholes is used as the main hole, and the hydraulic mining equipment is put into the borehole to implement borehole hydraulic mining for underground oil shale. This process mainly focuses on breaking oil shale with high-pressure water jet. In the meantime, the special hydraulic or pneumatic lifting equipment is put into another vertical borehole to pump the ore pulp of oil shale to the surface. In this way, the ore pulp of oil shale is produced in the process of the borehole hydraulic mining for underground oil shale cannot only be returned to the surface through the central hole of the hydraulic mining equipment, but also can be continuous pumped to the surface by the lifting equipment. This mining method is beneficial to ensure that the dynamic water table in the main hole is kept below the outlet of the jet devices, so it is easy to achieve the borehole hydraulic mining for underground oil shale in the non-submerged condition.

In addition, because the actual underground situation in the field is very complicated, the strength of the underground oil shale has a quite particularity in the process of the borehole hydraulic mining technology which is applied to the exploitation of the underground oil shale deposit. That also means the technological parameters of the actual borehole hydraulic mining for underground oil shale should be adjusted with the actual conditions of the oil shale deposit, and the adjustments should be made at any time to be suitable for the actual mining. Moreover, in order to obtain higher mining efficiency, the jet pressure and jet flow should be raised as much as possible under the preconditions of safety and reliability of the hydraulic mining equipment and pipelines connection.

The results and conclusions of this study can provide valuable guidance for borehole hydraulic mining of underground oil shale. Although, the experiments of this study are just to break the separated oil shale samples with high-pressure water jet, the crustal stress influences on the effect of breaking oil shale in the process of the actual borehole hydraulic mining for underground oil shale is neglected. The crustal stress includes overlying strata pressure and surrounding rock pressure. Therefore, the results and conclusions of this study may have some deviations from the actual mining situation. In addition, Wei Wei presents a mini-review about the geometrical fractal and hydraulic properties of fractured reservoirs, these methods are good approaches to solve the problem of crack propagation [33]. In future studies, the experimental device can be improved. The influences of the crustal stress on the broken oil shale with high-pressure water jet can be simulated by means of hydraulic or pneumatic loading. And the process of high pressure water jet breaking oil shale can also be tried to use the fractal method to research.

## Conclusions

1. Different water jet types have their own characteristics. The jet performance of the non-submerged water jet is better than the submerged water jet. Similarly, different water jet devices have their own optimal structural parameters. Each kind of jet devices should follow its basic principle and design principle. In addition, the self-developed multifunctional experimental device can fully meet the requirements of the experimental research conditions for testing the water jet performances of jet devices and breaking oil shale with high-pressure water jet.
2. In the case of the non-submerged condition, the water jet performance of the self-excited oscillation pulsed jet nozzle is always better than the straight cone nozzle. Moreover, the striking force of the direct water jet which is produced by straight cone nozzle increases with the jet distance increasing, and then its striking force decreases with the jet distance increasing. The optimal jet distance of the straight cone nozzle is fifty times the diameter of its outlet. Furthermore, the striking force of the pulsed water jet which is produced by the self-excited oscillation pulsed jet nozzle decreases with the jet distance increasing. In addition, for the relationship curve of the water jets’ striking force and jet distance, the trend curves and the experimental curves can be highly fitted, and these curves can be described by the polynomial equations or exponential equations.
3. No matter what kind of jet devices are used, it is always easier to break oil shale when the high-pressure water jets are in the parallel bedding direction of the oil shale. Also, it is easier to break oil shale when microcrack or crack existed. Moreover, the pulsed water jet which is produced by the self-excited oscillation pulsed jet nozzle is the best way to break oil shale.
4. The better parameters of borehole hydraulic mining for oil shale are as follows: the operating condition is non-submerged, the jet device is the self-excited oscillation pulsed jet nozzle which can produce the pulsed water jet, the jet direction is parallel to the oil shale bedding, the jet pressure is not less than 8 MPa, the jet flow is not less than 7.69 m^3^/h. In addition, to obtain the higher mining efficiency under the preconditions of ensuring the safety and reliability of the hydraulic mining equipment and pipelines connection, the jet pressure and jet flow should be raised as much as possible.

## Supporting information

S1 Table Technical parameters of the high-pressure pump in the self-developed multifunctional experimental device. (DOC)

S2 Table Technical parameters of the pressure transmitter in the self-developed multifunctional experimental device. (DOC)

S3 Table Technical parameters of the Intelligent turbine flowmeter in the self-developed multifunctional experimental device. (DOC)

S4 Table Technical parameters of the weighing sensor in the self-developed multifunctional experimental device. (DOC)
