# Supplementary material for: Experimental research on the performances of water jet devices and proposing the parameters of borehole hydraulic mining for oil shale
Source: PLoS One. 2018 Jun 20;13(6):e0199027. doi: 10.1371/journal.pone.0199027 (PMC6010288; doi:10.1371/journal.pone.0199027)
Supplement: S1 Table — (DOC) [file pone.0199027.s001.doc]

**S1 Table. Technical parameters of the high-pressure pump in the self-developed multifunctional experimental device.**

| **Serial Number** | **Items** | **Parameter Values** |
| --- | --- | --- |
| 1 | Product Model | 3D2-S |
| 2 | Motor Power, kW | 90 |
| 3 | Rated Pressure, MPa | 21 |
| 4 | Rated Flow, L/min | 215 |
| 5 | Pump Speed, Times per min | 500 |
| 6 | Plunger Quantity | 3 |
| 7 | Plunger Diameter, mm | 45 |
| 8 | Plunger Stroke, mm | 95 |
| 9 | Input Shaft Rotational Speed, r/min | 1480 |
| 10 | Weight, kg | 331 |
