# Supplementary material for: Experimental research on the performances of water jet devices and proposing the parameters of borehole hydraulic mining for oil shale
Source: PLoS One. 2018 Jun 20;13(6):e0199027. doi: 10.1371/journal.pone.0199027 (PMC6010288; doi:10.1371/journal.pone.0199027)
Supplement: S2 Table — (DOC) [file pone.0199027.s002.doc]

**S2 Table. Technical parameters of the pressure transmitter in the self-developed multifunctional experimental device.**

| **Serial Number** | **Items** | **Parameter Values** |
| --- | --- | --- |
| 1 | Product Model | LK-062 |
| 2 | Measure Range, MPa | 0 to 20 |
| 3 | Operating Pressure, MPa | ≤20 |
| 4 | Intrinsic Error | ±0.5% |
| 5 | Power Supply Mode | 24 V DC |
| 6 | Output Signal, mA | 4 to 20 |
| 7 | Operating Ambient Temperature, ℃ | -20 to 85 |
| 8 | Threaded Interface | M 20×1.5 |
