# Supplementary material for: Experimental research on the performances of water jet devices and proposing the parameters of borehole hydraulic mining for oil shale
Source: PLoS One. 2018 Jun 20;13(6):e0199027. doi: 10.1371/journal.pone.0199027 (PMC6010288; doi:10.1371/journal.pone.0199027)
Supplement: S4 Table — (DOC) [file pone.0199027.s004.doc]

**S4 Table. Technical parameters of the weighing sensor in the self-developed multifunctional experimental device.**

| **Serial Number** | **Items** | **Parameter Values** |
| --- | --- | --- |
| 1 | Product Model | CXL-102 |
| 2 | Measure Range, kg | 0 to 200 |
| 3 | Intrinsic Error | ±0.1% |
| 4 | Power Supply Mode | 24 V DC |
| 5 | Output Signal, mA | 4 to 20 |
| 6 | Operating Ambient Temperature, ℃ | -20 to 60 |
| 7 | Threaded Interface | M 16×1.5 |
